# The emerging spectrum of COVID-19 neurology: clinical, radiological and laboratory findings

**DOI:** 10.1093/brain/awaa240

**Published:** 2020-07-08

**Authors:** Ross W Paterson, Rachel L Brown, Laura Benjamin, Ross Nortley, Sarah Wiethoff, Tehmina Bharucha, Dipa L Jayaseelan, Guru Kumar, Rhian E Raftopoulos, Laura Zambreanu, Vinojini Vivekanandam, Anthony Khoo, Ruth Geraldes, Krishna Chinthapalli, Elena Boyd, Hatice Tuzlali, Gary Price, Gerry Christofi, Jasper Morrow, Patricia McNamara, Benjamin McLoughlin, Soon Tjin Lim, Puja R Mehta, Viva Levee, Stephen Keddie, Wisdom Yong, S Anand Trip, Alexander J M Foulkes, Gary Hotton, Thomas D Miller, Alex D Everitt, Christopher Carswell, Nicholas W S Davies, Michael Yoong, David Attwell, Jemeen Sreedharan, Eli Silber, Jonathan M Schott, Arvind Chandratheva, Richard J Perry, Robert Simister, Anna Checkley, Nicky Longley, Simon F Farmer, Francesco Carletti, Catherine Houlihan, Maria Thom, Michael P Lunn, Jennifer Spillane, Robin Howard, Angela Vincent, David J Werring, Chandrashekar Hoskote, Hans Rolf Jäger, Hadi Manji, Michael S Zandi

**Affiliations:** a1 University College London, Queen Square Institute of Neurology, London, UK; a2 Darent Valley Hospital, Dartford, Kent, UK; a3 UK Dementia Research Institute, London, UK; a4 UCL Institute of Immunity and Transplantation, London, UK; a5 Stroke Research Centre, UCL Queen Square Institute of Neurology, London, UK; a6 University of Liverpool, Brain Infections Group, Liverpool, Merseyside, UK; a7 Wexham Park Hospital, Frimley Health NHS Foundation Trust, Berkshire, UK; a8 Center for Neurology and Hertie Institute for Clinical Brain Research, Eberhard-Karls-University, Tübingen, Germany; a9 National Hospital for Neurology and Neurosurgery, University College London Hospitals NHS Foundation Trust, Queen Square, London, UK; a10 Department of Biochemistry, University of Oxford, Oxford, UK; a11 Lao-Oxford-Mahosot Hospital-Wellcome Trust-Research Unit, Mahosot Hospital, Vientiane, Laos; a12 Watford General Hospital, Watford, Hertfordshire, UK; a13 King’s College Hospital, Denmark Hill, London, UK; a14 University of Oxford, Nuffield Department of Clinical Neurosciences, John Radcliffe Hospital, Oxford, UK; a15 Northwick Park Hospital, Harrow, London, UK; a16 Lister Hospital, Stevenage, Hertfordshire, UK; a17 Imperial College Healthcare NHS Trust, London, UK; a18 Chelsea and Westminster Hospital, London, UK; a19 Barts and The London NHS Trust, London, UK; a20 UCL, Department of Neuroscience, Physiology and Pharmacology, London, UK; a21 Hospital for Tropical Diseases, University College London Hospitals NHS Foundation Trust, London, UK; a22 Lysholm Department of Neuroradiology, National Hospital for Neurology and Neurosurgery, University College London Hospitals NHS Foundation Trust, Queen Square, London, UK; a23 UCL Division of Infection and Immunity, London, UK; a24 Guy's and St Thomas’ NHS Foundation Trust, London, UK

**Keywords:** COVID-19, SARS-CoV-2, encephalitis, ADEM

## Abstract

Preliminary clinical data indicate that severe acute respiratory syndrome coronavirus 2 (SARS-CoV-2) infection is associated with neurological and neuropsychiatric illness. Responding to this, a weekly virtual coronavirus disease 19 (COVID-19) neurology multi-disciplinary meeting was established at the National Hospital, Queen Square, in early March 2020 in order to discuss and begin to understand neurological presentations in patients with suspected COVID-19-related neurological disorders. Detailed clinical and paraclinical data were collected from cases where the diagnosis of COVID-19 was confirmed through RNA PCR, or where the diagnosis was probable/possible according to World Health Organization criteria. Of 43 patients, 29 were SARS-CoV-2 PCR positive and definite, eight probable and six possible. Five major categories emerged: (i) encephalopathies (*n *=* *10) with delirium/psychosis and no distinct MRI or CSF abnormalities, and with 9/10 making a full or partial recovery with supportive care only; (ii) inflammatory CNS syndromes (*n *=* *12) including encephalitis (*n *=* *2, para- or post-infectious), acute disseminated encephalomyelitis (*n *=* *9), with haemorrhage in five, necrosis in one, and myelitis in two, and isolated myelitis (*n *=* *1). Of these, 10 were treated with corticosteroids, and three of these patients also received intravenous immunoglobulin; one made a full recovery, 10 of 12 made a partial recovery, and one patient died; (iii) ischaemic strokes (*n *=* *8) associated with a pro-thrombotic state (four with pulmonary thromboembolism), one of whom died; (iv) peripheral neurological disorders (*n *=* *8), seven with Guillain-Barré syndrome, one with brachial plexopathy, six of eight making a partial and ongoing recovery; and (v) five patients with miscellaneous central disorders who did not fit these categories. SARS-CoV-2 infection is associated with a wide spectrum of neurological syndromes affecting the whole neuraxis, including the cerebral vasculature and, in some cases, responding to immunotherapies. The high incidence of acute disseminated encephalomyelitis, particularly with haemorrhagic change, is striking. This complication was not related to the severity of the respiratory COVID-19 disease. Early recognition, investigation and management of COVID-19-related neurological disease is challenging. Further clinical, neuroradiological, biomarker and neuropathological studies are essential to determine the underlying pathobiological mechanisms that will guide treatment. Longitudinal follow-up studies will be necessary to ascertain the long-term neurological and neuropsychological consequences of this pandemic.

## Teaser

Paterson, Brown *et al.* present a case series of 43 patients with neurological complications of SARS-CoV-2 infection. The neurological syndromes include encephalopathies, encephalitis, acute disseminated encephalomyelitis with haemorrhagic change, transverse myelitis, ischaemic stroke, and Guillain-Barré syndrome.

## Introduction

Since December 2019, almost 10 million cases and 500 000 deaths due to the novel coronavirus, severe acute respiratory syndrome coronavirus 2 (SARS-CoV-2), have been reported worldwide (WHO situation report). Although the respiratory system complications of coronavirus disease 19 (COVID-19) have been the most frequent and life threatening, there are increasing reports of central and peripheral nervous system (PNS) involvement. These neurological complications have included encephalopathy (Helms *et al.*, 2020), meningo-encephalitis (Moriguchi *et al*., 2020), ischaemic stroke (Beyrouti *et al.*, 2020), acute necrotizing encephalopathy (Poyiadjin *et al*., 2020), and Guillain-Barré syndrome (GBS) (Toscano *et al.*, 2020). Radiological series have shown infarcts, microhaemorrhages, features of posterior reversible encephalopathy syndrome, or nerve root enhancement (Franceschi *et al*., 2020; Mahammedi *et al*., 2020). Zanin and colleagues (2020) have described a case of CNS demyelination post-COVID-19. Detailed neurological assessment and investigation is challenging in those who are critically ill, limiting the opportunity to delineate the underlying pathophysiology and hence, treatment options. The postulated mechanisms of the various neurological syndromes include, either individually or in combination, direct viral neuronal injury (Zubair *et al.*, 2020), a secondary hyperinflammation syndrome (Mehta *et al.*, 2020), para- and post-infectious inflammatory or immune-mediated disorders, or the effects of a severe systemic disorder with the neurological consequences of sepsis, hyperpyrexia, hypoxia, hypercoagulability and critical illness.

Here we describe the detailed emerging spectrum of neurological disorders encountered in 43 COVID-19 patients referred to the National Hospital, Queen Square COVID-19 multidisciplinary team meeting (COVID-MDT), run in partnership with infectious disease and virology colleagues at University College London Hospital (UCLH).

## Materials and methods

We reviewed retrospectively the clinical, radiological, laboratory and neuropathological findings from patients referred to the COVID-MDT neurology/encephalitis and neurovascular multi-disciplinary team meetings. The cases summarized were discussed between 9 April and 15 May 2020. Neurological syndromes developing after definite, probable or possible COVID-19, which were likely to be associated with COVID-19 on clinical grounds, were included. Cases for which a more likely alternative pathology was found were excluded.

The probability of COVID-19-related neurological disease was determined using WHO criteria [‘Global surveillance for human infection with coronavirus disease (COVID-19)’]: (i) definite (SARS-CoV-2 RNA PCR positive from nasopharyngeal swab, CSF or pathological specimen); (ii) probable (clinical and laboratory features highly suggestive of COVID-19: lymphopenia, raised D-dimer, suggestive chest radiology in the absence of PCR evidence) (Guan *et al.*, 2020); and (iii) possible, in whom temporal or laboratory features indicate an association but another cause was also found (Ellul *et al*., 2020). 

The classification of the severity of COVID-19 infection was adapted from Wu and McGoogan (2020). Mild disease included patients with non-pneumonia or mild pneumonia, severe disease included patients with dyspnoea and hypoxia requiring supplementary oxygen, and critical disease included patients with respiratory failure requiring assisted ventilation, septic shock, and/or multi-organ dysfunction. Where possible, laboratory results shown are those nearest to onset of neurological symptoms. Consensus clinical criteria were used to classify individuals with specific neurological syndromes including encephalitis (Solomon *et al*., 2012; Graus *et al*., 2016), acute demyelinating encephalomyelitis (ADEM) (Pohl *et al.*, 2016), and GBS (Willison *et al.*, 2016). We obtained assent and/or written consent from patients or from their relatives. This on-going study is approved and registered as a service evaluation of our MDT (ref 06–202021-SE) at University College London Hospitals NHS Trust.

Some patient details have been submitted for publication as case reports by their treating physicians: Patient 7 (Lim *et al.*, submitted for publication), Patient 11 (Khoo *et al*., 2020), Patient 12 (Zambreanu *et al*., 2020), Patient 15 (Dixon *et al.*, 2020), Patients 23, 24, 26, 28, 29 (Beyrouti *et al.*, 2020), and Patient 41 (Wilson *et al*., 2020).

### Data availability

The data that support the findings of this study are available from the corresponding author, upon reasonable request. The data are not publicly available due to ethical restrictions e.g. their containing information that could compromise the privacy of the patients reported.

## Results

The patients included 24 males and 19 females with ages ranging from 16 to 85 years. Twenty-three of our patients (53%) were non-white. Based on a positive nasal-pharyngeal throat SARS-CoV-2 PCR test, 29 were defined as definite COVID-19, eight were probable and six were possible for this association. The severity of the COVID-19 symptoms varied from mild to critical. The patients presented with a wide range of CNS and PNS features including neuroinflammatory diseases and stroke from 6 days before and up to 27 days following the onset of the COVID-19 symptoms. The patients are divided into five categories based on their clinical, neuroradiological, neurophysiological and laboratory features, as summarized in Table 1. We provide a brief summary of the neurological phenotypes in Tables 2–4. Full details of the clinical, viral, immunological, radiological and neurophysiological investigations, management and treatment responses are detailed in the Supplementary material.

**Table 1:** Summary of clinical features of 43 patients with neurological complications of COVID-19

Cases	Age, median [range]; %male	Days of COVID-19 infection before neurological presentation, median [range]	Main clinical features	Results of note	% Naso- pharyhgeal SARS-CoV-2 PCR+	CSF or brain SARS-CoV-2 PCR+ (*x*/number tested)	Treatment	Clinical outcome
Encephalopathy (delirium/psychosis) (*n =* 10)^a^	57.5 [39–72]; 40	4.5 [−4 to +21]	Delirium; psychosis	Acellular CSF (6/6); non-specific MRI changes (3/10)	80 (8/10)	(0/0)	Supportive (9/10); steroids 1/10	Complete recovery (7/10); partial (2/10)
Inflammatory CNS syndromes (para-/post- infectious) (*n =* 12)^a^	53 [27–66]; 33	9 [−6 to +27]	Reduced consciousness (7/12); UMN signs (10/12)	Abnormal CSF (6/11) Abnormal MRI (11/12)	67 (8/12)	(0/7)	Corticosteroids (10/12); IVIG (3/12)	Recovery: complete (1/12); partial (10/12); none (death 1/12)
Stroke (*n =* 8)^a^	62.5 [27–85]; 75	8[−2 to +22]	Large vessel ischaemic stroke	4/8 PE 6/6 High D–dimer	75 (6/8)	NA	Low molecular weight heparin (7/8); apixaban (1/8)	Incomplete recovery (7/8); death (1/8)
Peripheral syndromes (*n =* 8)								
GBS (*n =* 7)	57 [20–63]; 100	13 [−1 to +21]	Cranial and peripheral neuropathy		43 (3/7)	NT	IVIG (7/7)	Incomplete recovery (5/7 GBSDS 2)
Plexopathy (*n =* 1)	60; 100	14	Painless weakness		100 (1/1)	NT	IV steroids (1/1)	Incomplete recovery (1/1)
Miscellaneous and uncharacterized (*n =* 5)	20 [16–40]; 40	10 [+6 to +26]	Raised ICP; seizures; myelitis	Abnormal CSF (2/4) Abnormal MRI brain (4/5)	60 (3/5)	(0/1)	Varied (AED; steroids (1/5); tLP)	Recovery complete (1/5); partial (3/5); nil (1/5)

AED = anti-epileptic drug; GBSDS = Guillain Barré disability score; ICP = intracranial pressure; tLP = therapeutic lumbar puncture; NT = not tested; PE = pulmonary thromboembolism; UMN = upper motor neuron.

a Features of eight individual patients for encephalopathy (delirium/psychosis), inflammatory CNS syndromes (para/post-infectious) and stroke described in Tables 2–4. All patient details are available in the Supplementary material.

**Table 2:** Eight patients with spontaneously improving encephalopathies (Patients 1–8)

Patient	1	2	3	4	5	6	7	8
Age, M/F, ethnicity, COVID-19 diagnosis/severity	65, F, White, definite/mild	72, M, White, definite/critical	59, F, Black, definite/mild	58, M, Black, definite/mild	52, F, White, probable/mild	39, F, Asian, definite/critical	55, F, White, definite/severe	68, M, Black, definite/mild
Final neurological diagnosis	Hypoactive delirium	Hypoactive delirium	Delirium	Delirium	Delirium	Delirium	Delirium and psychosis	Hyperactive delirium
Initial neurological symptoms	Fluctuating confusion; reversal of sleep-wake cycle	Confusion; malaise; loss of appetite	Fluctuating confusion	Confusion; nonsensical speech; repetitive behaviour; disorientation; delusional thoughts; headache	Fluctuating consciousness; delirium	Delirium; hallucinations about experiences in countries not previously visited; reversed sleep/wake cycle	Confusion; agitation; persecutory delusions; visual hallucinations; combative behaviour; headaches	Cognitive impairment; gait disturbance; two falls
Key neurological signs	Disorientated to time and place; impaired insight; bradyphrenia; polyminimyoclonus; old left homonymous hemianopia	Cognitive impairment; increased limb tone; brisk reflexes	Fluctuating attention and cognition; bradyphrenia; dyspraxia.	Bilateral intention tremor; heel-shin ataxia	Cognitive impairment; reduced verbal fluency	Cognitive impairment	No focal signs	Disorientation; intermittent agitation; unable to follow commands; speaking a few words only; bilateral extensor plantars
D-dimer (µg/l; 0–550)	1190	1730	NR	970	NR	2430	1200	NR
Neurological treatments; recovery	Supportive; complete	Supportive; complete/rehab	Supportive; complete	Supportive; complete	Supportive; complete	Melatonin; on-going cognitive impairment	Haloperidol, risperidone; improving	1g IVMP 3 days; ongoing improvement

Imaging, further investigations and Patients 9 and 10 are provided in the Supplementary material. F = female; IVMP = intravenous methylprednisolone; M = male; NR = no result.

**Table 3:** Eight patients with neuroinflammatory diseases (Patients 11–18)

Patient	11	12	13	14	15	16	17	18
Age, M/F, ethnicity, COVID-19 diagnosis/ severity	65, F, Black, definite/severe	66, F, White, definite/mild	52, M, Asian, definite/critical	60, M, Asian, definite/critical	59, F, Asian, definite/mild	52, M, White, definite/critical	47, F, other, probable/severe	54, F, mixed, probable/mild
Final neurological diagnosis	Possible post-infectious encephalitis (presumed autoimmune)	Encephalitis	ADEM (with haemorrhage)	ADEM (with haemorrhage)	ADEM (with necrosis and haemorrhage)	ADEM (with haemorrhage) and acute demyelinating polyradiculoneuropathy	ADEM (with haemorrhage)	ADEM
Imaging: neuraxis (summary)	MRI brain normal	MRI brain: T_2_ hyperintense signal changes in upper pons, limbic lobes, medial thalami and subcortical cerebral white matter	MRI brain: multiple clusters of lesions in the deep cerebral white matter. Cyst-like areas of varied sizes, some with haemorrhagic foci and peripheral rims of restricted diffusion	MRI brain: multifocal and confluent areas of signal change in the cerebral hemispheric white matter with extensive microhaemorrhages in the subcortical regions	MRI brain (Day 6): extensive, confluent and largely symmetrical areas throughout brainstem, limbic and insular lobes, superficial subcortical white matter and deep grey matter. Clusters of microhaemorrhages, restricted diffusion and peripheral rim enhancement	MRI brain: multifocal confluent lesions in internal and external capsules, splenium and deep white matter of cerebral hemispheres. Over 5 days, lesions increased in size and showed multiple microhaemorrhages and extensive prominent medullary veins. Components of brachial and lumbosacral plexus showed increased signal and enhancement	Severe right hemispheric vasogenic oedema with a leading edge on contrast imaging. Smaller areas of T_2_ hyperintense changes in the left hemisphere. Marked mass-effect with 10 mm leftwards midline shift, and mild subfalcine herniation	Multiple large lesions with peripheral rim restriction in periventricular white matter of both cerebral hemispheres
D-dimer if raised; CSF studies; all neuronal antibodies performed were negative	1800 µg/l (0–550); CSF matched OCB, viral PCR negative	1599 ng/ml (0–230); CSF protein raised, OCB, viral PCR including SARS-CoV-2 negative	80 000 µg/l (0–550); OCB negative, viral PCR and antibodies negative	3330 mg/l (250–750); CSF OCB negative, viral PCR negative including SARS-CoV-2	2033 mg/l (250–750); CSF OP raised, viral PCR negative including SARS-CoV-2	NR; CSF protein raised, viral PCR negative	1160 µg/l (0–550); CSF NR	NR 19 (90% lymph); 0.33; OCB negative, CSF culture scanty *Staphylococcus capitis* (likely contamination)
Treatments for neurological diagnosis; recovery	1 g IVMP 3 days, oral prednisolone taper, levetiracetam, clonazepam; incomplete	1 g IVMP 3 days then oral prednisolone taper, IVIG; incomplete	Supportive; incomplete but ongoing	1 g IVMP 3 days; incomplete ongoing	Intubation, ventilation; levetiracetam, aciclovir and ceftriaxone, dexamethasone; no response, died	Intubation and ventilation, 1 g IVMP 5 days, IVIG; incomplete ongoing recovery	Intubation, hemicraniectomy, 1g IVMP 5 days, oral prednisolone, IVIG; incomplete ongoing recovery	1 g IVMP 3 days, then oral prednisolone; incomplete ongoing recovery

Diagnosis, imaging and further investigations for Patients 19–22 are provided in the Supplementary material. F = female; IVMP = intravenous methylprednisolone; M = male; NR = no result; OCB = oligoclonal band; OP = opening pressure.

**Table 4:** Eight patients with stroke (Patient 23–30)

Patient	23	24	25	26	27	28	29	30
Age, M/F, ethnicity, COVID-19 diagnosis, severity, time from COVID onset	61, M, Black, definite/mild, 2 days	64, M, White, definite/severe , 15 days	64, M, White definite/severe, NK	53, F, Asian, definite/severe, 22 days	58, M, Black, probable/mild, 2 days	85, M, White, definite/mild, 10 days	73, M, Asian, definite/mild, 8 days	27, F, White, probable/mild, 0 days
Stroke type, observed/implicated mechanism; venous thromboembolism	Ischaemic right middle cerebral artery occlusion; yes, PE	Ischaemic, vertebral-basilar artery occlusion; yes, PE	Ischaemic bilateral ACA-MCA and MCA-PCA cortical and deep borderzone infarct; no	Ischaemic, vertebral-basilar artery occlusion; no	Ischaemic, proximal left middle cerebral artery occlusion; yes PE	Ischaemic, left posterior cerebral artery occlusion; no	Ischaemic basilar artery occlusion, no	Ischaemic left internal cerebral artery occlusion; yes PE
Fibrinogen (g/l; 1.5–4.0), D-dimer (µg/l; 0–550), Prothrombin time (s; 10–12)	4.63, 27 190, 10.7	9.5, 80 000, 11.6	8.82, 29 000, 12.6	2.91, 7750, 34.4	3.15, 75 320, 12,2	5.3, 16 100, 11.3	NR, NR, 14.9	NR, NR, 11.5
Brain imaging (summary)	MRI: acute infarct in the right corpus striatum. Multiple supra- and infra-tentorial cortical and subcortical microhaemorrhages	MRI: (1st event): acute left vertebral artery thrombus and acute left posterior-inferior cerebellar artery territory infarction with microhaemorrhages. 2nd event, 7 days later: bilateral acute posterior cerebral artery territory infarcts despite therapeutic anticoagulation	MRI: subacute infarcts within the deep internal border zones of the cerebral hemispheres bilaterally, and within the left frontal white matter. Background moderate small vessel disease and established cortical infarcts, in arterial border zone territories	Non-contrast CT: showed acute right parietal cortical and left cerebellar infarct with mass effect and hydrocephalus, despite therapeutic anticoagulation	MRI: extensive evolving left MCA infarct with evidence of petechial haemorrhage and associated mass-effect as described. Persistent occlusion of the left M2 MCA branches	Non-contrast CT: showed hyperdensity consistent with thrombus in the left posterior cerebral artery and acute infarction in the left temporal stem and cerebral peduncle	MRI: acute infarction in the right thalamus, left pons, right occipital lobe and right cerebellar hemisphere	CT: right middle cerebral artery and right anterior cerebral artery territory infarction
Tissue plasminogen activator, mechanical ventilation, anti-thrombotic therapy	No, no, LMWH	No, no, LMWH	No, no, LMWH	No, no, LMWH	No, no, LMWH	No, no, aspirin 7 days then switched to apixaban	Yes, no, aspirin 5 days then switched to LMWH	Aspirin 10 days then LMWH
Outcome status	Rehabilitation unit	Rehabilitation unit	Remains static in ICU (Day 31)	Died	Rehabilitation unit	Rehabilitation unit	Stroke unit	Rehabilitation unit

ACA = anterior cerebral artery F = female; ICU = intensive care unit; LMWH = low molecular weight heparin; M = male; MCA = medial cerebral artery; NK = not known; NR = no result; PCA = posterior cerebral artery; PE = pulmonary embolism.

### CNS syndromes

#### Encephalopathies

The 10 patients described (Patients 1–10, six female, four male; four White, five Black and one Asian) had a para-infectious or septic encephalopathy with delirium. These patients (e.g. Vignette A) were mostly >50 years old and presented with confusion and disorientation, with psychosis in one, and seizures in another. Neuroimaging was within normal limits, and CSF studies were normal when performed. Treatments were largely supportive with 7 of 10 making a complete recovery, and 2 of 10, a partial recovery at the time of discharge (Table 2 and Supplementary material).

#### Vignette A: acute para-infectious encephalopathy with psychosis

A 55-year-old female (Patient 7), with no previous psychiatric history, was admitted with a 14-day history of fever, cough, muscle aches, breathlessness, as well as anosmia and hypogeusia. She required minimal oxygen treatment (oxygen saturation 94% on room air) and was well on discharge 3 days later. The following day, her husband reported that she was confused and behaving oddly. She was disorientated and displayed ritualistic behaviour such as putting her coat on and off repeatedly. She reported visual hallucinations, seeing lions and monkeys in her house. She developed ongoing auditory hallucinations, persecutory delusions, a Capgras delusion and complex systematized delusional misperceptions. She displayed intermittently aggressive behaviour with hospital staff and her family. Her psychotic symptoms persisted after disorientation improved. Brain MRI, EEG and lumbar puncture were normal. Her clinical course fluctuated over 3 weeks with a trend towards improvement, albeit after the introduction of haloperidol, followed by risperidone.

### Neuroinflammatory syndromes

**Figure 1:**
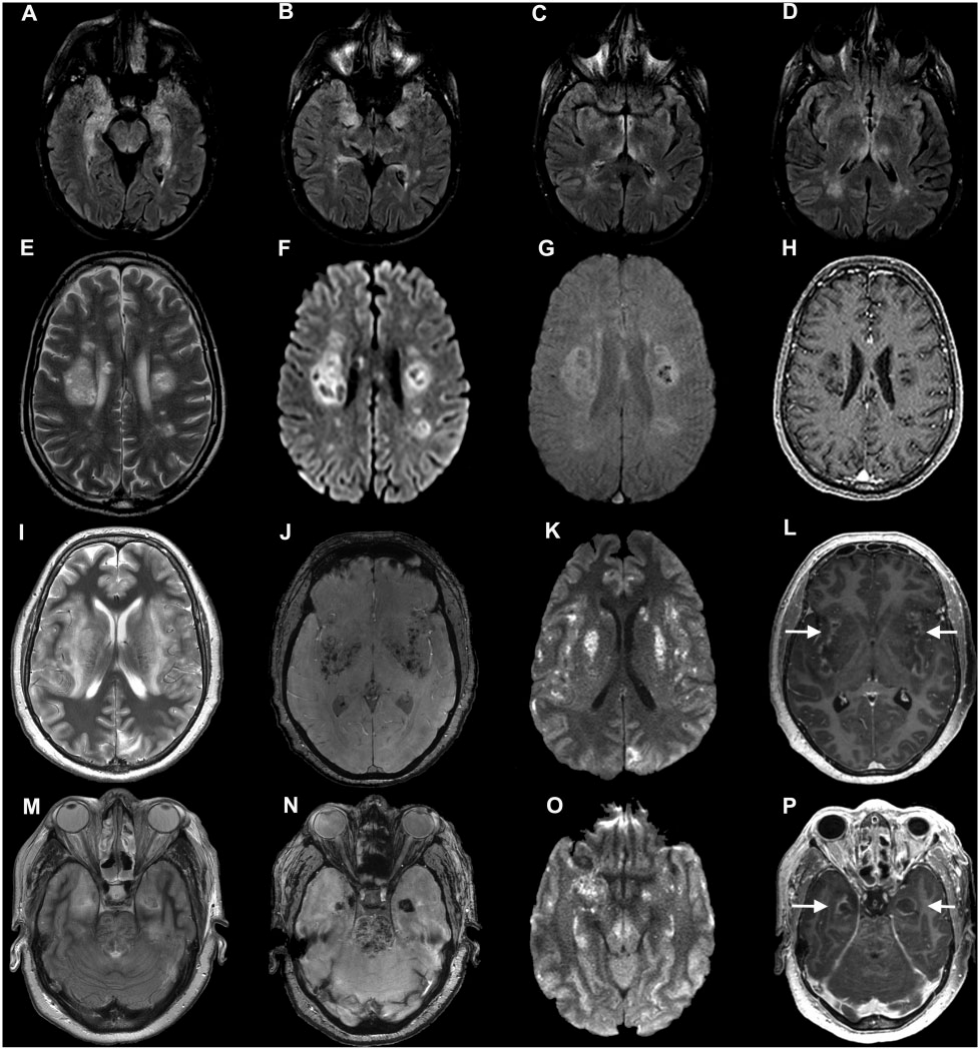
**Imaging from Patients 12, 13 and 15 (COVID-19 autoimmune and haemorrhagic encephalitis).** Axial MRI from three individuals with para-/post-infectious central syndromes. (**A**–**D**) Patient 12: axial fluid-attenuated inversion recovery (FLAIR) images show bilateral hyperintensity in the mesial temporal lobes (**A** and **B**), hypothalamus (**C**) temporal lobes and thalamus (**D**). (**E**–**H**) Patient 13: axial T_2_-weighted (**E**), diffusion weighted imaging (DWI) (**F**), susceptibility weighted imaging (SWI) (**G**) and post-contrast T_1_-weighted (**H**) images show multifocal clusters of lesions involving the deep white matter of both cerebral hemispheres, intralesional cyst-like areas of varied sizes, and some peripheral rims of restricted diffusion (**F**), some haemorrhagic changes (**G**), and T_1_ hypointense ‘black holes’ without contrast enhancement (**H**). (**I**–**P**) Patient 15: axial images at the level of the insula and basal ganglia (**I**–**L**) and at the level of the temporal lobes and upper pons (**M**–**P**). T_2_-weighted images (**I** and **M**), SWI images (**J** and **N**), DWI images (**K** and **O**) and contrast-enhanced images (**L** and **P**). There are extensive confluent areas of T_2_ hyperintensity (**I** and **M**), with haemorrhagic change on SWI imaging (**J** and **N**), restricted diffusion on DWI images (**K** and **O**) and peripheral contrast-enhancement (arrows in **L** and **P**) in the insular region, basal ganglia and left occipital lobe (**I**–**L**) as well as in the medial temporal lobes and upper pons (**M**–**P**).

Twelve patients (Patients 11–22, 27–66 years old; eight female, four male; four White, three Black, three Asian; two other/mixed) presented with inflammatory CNS syndromes. Two had an encephalitis; one (Patient 11, Vignette B) had features of an autoimmune encephalitis with opsoclonus, stimulus sensitive myoclonus and convergence spasm. Although brain imaging, EEG and CSF were normal, the clinical picture was highly suggestive of an autoimmune brainstem encephalitis. The second encephalitis patient (Patient 12) presented with confusion and a single seizure, with MRI abnormalities suggestive of autoimmune or ‘limbic’ encephalitis in the thalami, medial temporal regions and pons (Fig. 1A–D, Table 3 and Supplementary material).

**Figure 2:**
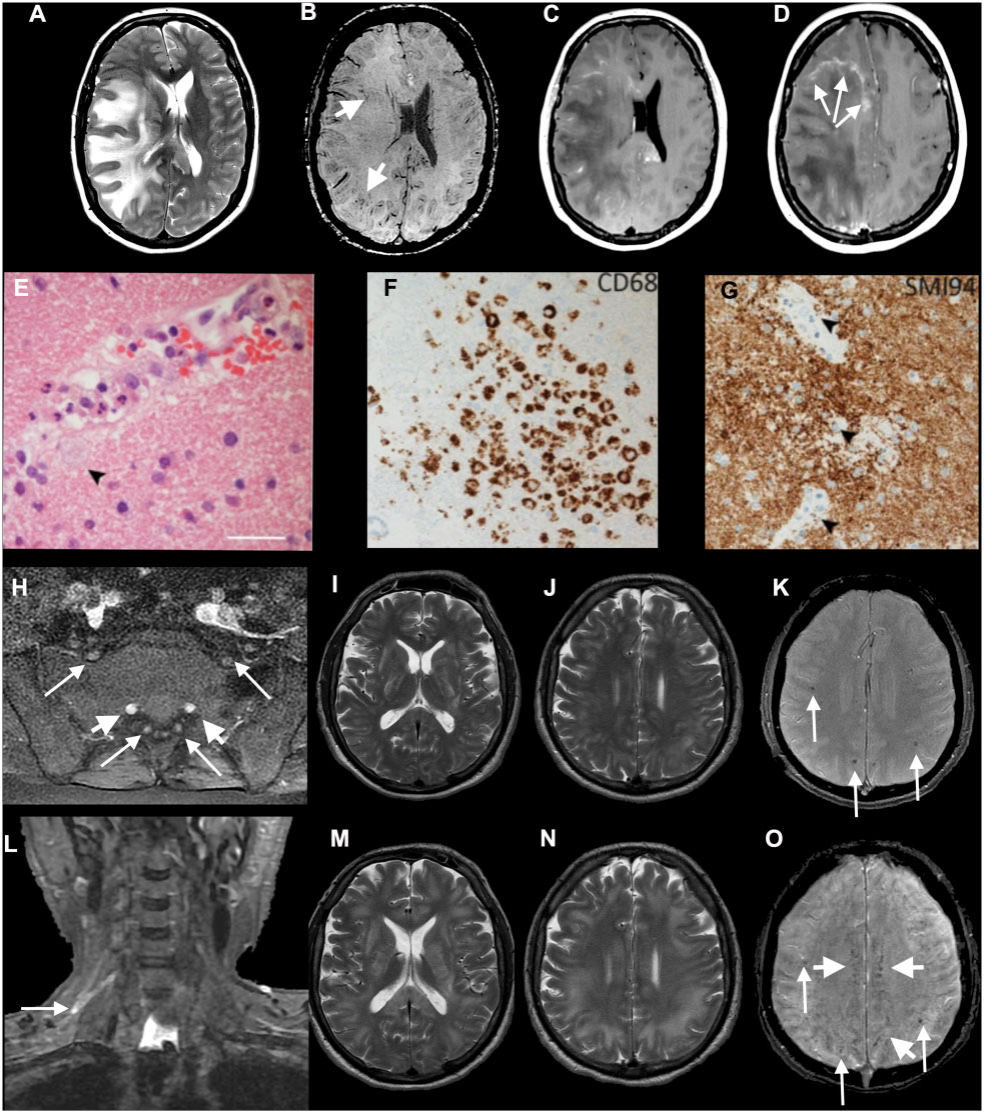
**Axial MRI (A–D) and histopathology (E–G) from Patient 17, diagnosed with ADEM, and imaging (H–O) from Patient 16, with combined CNS and PNS disease.** (**A**–**G**) Patient 17: axial T_2_-weighted (**A**), SWI (**B**), post-gadolinium (**C** and **D**) images show extensive confluent ‘tumefactive’ lesions involving the white matter of the right cerebral hemisphere, corpus callosum and corona radiata with mass effect, subfalcine herniation (**A**), clusters of prominent medullary veins (**B**, short arrows) and peripheral rim enhancement (**D**, arrows). (**E**) The white matter shows scattered small vessels with surrounding infiltrates of neutrophils and occasional foamy macrophages extending into the parenchyma (arrow). The endothelium is focally vacuolated but there is no evidence of vasculitis or fibrinoid vessel wall necrosis in any region. There were a few perivascular T cells in the white matter but the cortex appears normal (not shown). (**F**) CD68 stain confirms foci of foamy macrophages in the white matter, mainly surrounding small vessels. There was no significant microgliosis in the cortex (not shown). (**G**) Myelin basic protein stain (SMI94) shows areas with focal myelin debris in macrophages around vessels in the white matter (arrows) in keeping with early myelin breakdown. There is no evidence of axonal damage on neurofilament stain (not shown). Scale bars: **E **=** **45 µm; **F** and **G **=** **70 µm. (**H**–**O**) Patient 16: axial post-gadolinium fat-suppressed T_1_-weighted images (**H**) demonstrating pathologically enhancing extradural lumbosacral nerve roots (arrows). Note physiological enhancement of nerve root ganglia (short arrows). Coronal short tau inversion recovery (STIR) image (**L**) shows hyperintense signal abnormality of the upper trunk of the right brachial plexus (arrow). Initial axial T_2_ (**I** and **J**) and T_2_*-weighted images (**K**) show multifocal confluent T_2_ hyperintense lesions involving internal and external capsules, splenium of corpus callosum (**I**), and the juxtacortical and deep white matter (**J**), associated with microhaemorrhages (**K**, arrows). Follow-up T_2_-weighted images (**M** and **N**) show marked progression of the confluent T_2_ hyperintense lesions, which involve a large proportion of the juxtacortical and deep white matter, corpus callosum and internal and external capsules. The follow-up SWI image (**O**) demonstrates not only the previously seen microhaemorrhages (arrows) but also prominent medullary veins (short arrows).

Nine patients were categorized within the spectrum of ADEM (e.g. Vignette C, Fig. 1E–H). Four patients had haemorrhagic change on imaging, including microbleeds; and one had necrosis. Two patients had myelitis in addition to brain imaging changes, and one further had myelitis with normal brain imaging. Patient 17 (Vignette D) with acute haemorrhagic leucoencephalitis (based on clinical and imaging features) failed to respond to corticosteroids and required decompressive craniectomy for incipient brain herniation; a brain biopsy at the time of surgery showed evidence of perivenular inflammation supporting aggressive hyper-acute ADEM. She made significant recovery after the decompression followed by intravenous immunoglobulin (IVIG), but requires ongoing rehabilitation. Patient 15 developed a severe necrotizing encephalitis (Fig. 1I–P) that resulted in death. Patient 16 was unusual in presenting with a GBS and subsequently developed an ADEM-like illness (Fig. 2H–O, Vignette E).

**Figure 3:**
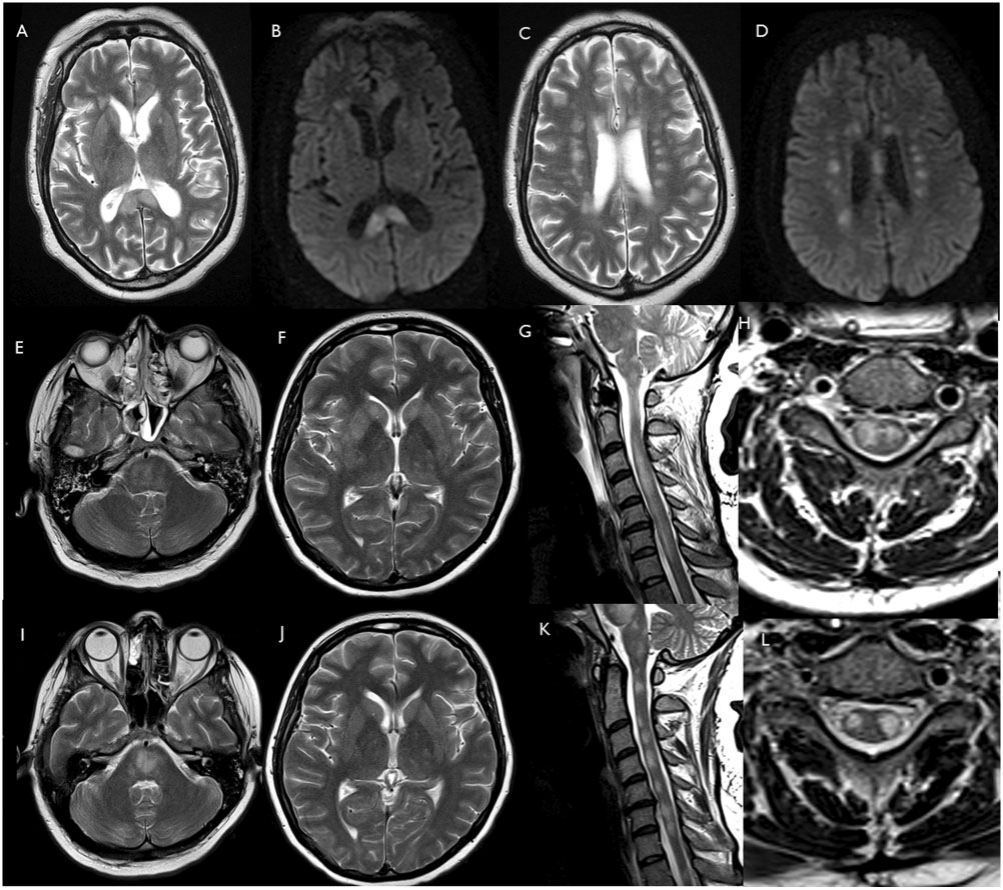
**Patients 19 and 20 (ADEM including spinal cord).** Patient 19: axial T_2_ (**A** and **C**) and DWI (**B** and **D**) images show multifocal lesions involving corpus callosum and corona radiata. Patient 20: axial T_2_-weighted images of brain MRI and sagittal T_2_-weighted of the spinal cord acquired on admission (**E**–**H**) and after 26 days (**I**–**L**). Axial T_2_-weighted images show multifocal hyperintense lesions in the brainstem (**E** and **I**), basal ganglia and supratentorial white matter (**F** and **J**). The pontomedullary hyperintensities have become more confluent (**I**) since admission (**E**). After 26 days, the signal abnormalities in the basal ganglia and the supratentorial white matter (**J**) are grossly similar to the baseline MRI scan (**F**). Sagittal and axial T_2_-weighted images show diffuse high T_2_-weighted signal intrinsic to the spinal cord at baseline (**G** and **H**). After 26 days, the cord oedema has reduced, and the spinal cord lesions appear less confluent and more discrete (**K** and **L**).

Despite the striking imaging findings of these patients (Figs 1–3), the CSF parameters were abnormal in only half. In none of the cases tested were specific antibodies (e.g. to NMDAR, MOG, AQP4, LGI1 or GAD) identified in the serum or CSF. Treatments were with corticosteroids in nine, and IVIG in three. A full clinical response was seen in 1 of 12, partial recovery at the time of writing in 10 of 12, and one patient died.

#### Vignette B: post-infectious probable brainstem and cortical autoimmune encephalitis

A 65-year-old female (Patient 11), with a 2-year history of cognitive decline and presumed sporadic early onset Alzheimer’s disease, presented with right hand and then widespread involuntary movements, 6 days after fever, cough and myalgia. She had difficulty speaking and became disorientated and confused, complaining of well-formed visual hallucinations of people inside her house and objects flying around the room. She complained of deteriorating vision, with difficulty reading, and intermittent double vision. On admission she had widespread stimulus sensitive myoclonus involving the tongue and all four limbs with marked hyperekplexia. There was episodic opsoclonus and prominent convergence spasm on visual fixation. She had a non-fluent aphasia with oral apraxia, difficulty repeating sentences and was only able to follow single stage commands. MRI brain, EEG and CSF examination were normal. SARS-CoV-2 PCR was positive on nasopharyngeal swab. Levetiracetam and clonazepam were used to treat her myoclonus, and 2 weeks after onset of neurological symptoms, she received a course of steroids for a clinical diagnosis of presumed post-infectious autoimmune encephalitis affecting cortex and brainstem. Cognition and visual symptoms improved although there are on-going symptoms at the time of writing.

#### Vignette C: ADEM with haemorrhage in a critically ill patient

A 52-year-old male (Patient 13) presented with a 10-day history of cough, fever, dyspnoea and myalgia. On admission he was hypoxic and non-invasive ventilation was commenced. He had bilateral chest X-ray changes consistent with COVID-19 and SARS-CoV-2 RNA PCR was positive. Oxygen requirements increased and mechanical intubation was required. On Day 17 of intensive care admission he was slow to wean from sedation. His conscious level was impaired (responding to pain only) despite a prolonged withdrawal from sedation. He was hyper-reflexic with lower limb clonus. Brain MRI showed bilateral white matter changes with haemorrhage (Fig. 1E–H). There was slow and still on-going neurological improvement over 4 weeks with supportive treatment alone, which continues at the time of writing.

#### Vignette D: acute haemorrhagic leukoencephalopathy form of ADEM requiring decompressive craniectomy

A 47-year-old female (Patient 17), previously well and who worked in a high-risk occupation for COVID-19, presented with right-sided headache and left hand numbness. This was preceded by 7 days of cough, fever and shortness of breath. On the day of presentation, she had persistent severe headache and progressive onset of left-sided numbness followed by left-sided weakness including the face. A few hours later, she was drowsy, with severe left upper limb weakness, mild left leg weakness and hemisensory loss. CT head imaging demonstrated marked right hemisphere vasogenic oedema with midline shift. She required 4 l of oxygen and had lower zone chest X-ray and CT chest changes compatible with probable COVID-19 as well as lymphopenia, and elevated D-dimer. Head MRI demonstrated severe right hemispheric vasogenic oedema with a leading edge on contrast imaging, and smaller areas of T_2_ hyperintense changes in the left hemisphere, in keeping with a diagnosis of an acute haemorrhagic leukoencephalopathy form of ADEM. She was treated with high dose intravenous methylprednisolone (1 g daily for 5 days). After 48 h of treatment her conscious level fell, she developed a fixed dilated right pupil and underwent emergency right hemi-craniectomy. She subsequently received oral prednisolone 60 mg daily and 5 days of IVIG. She was extubated 4 days postoperatively and continues to improve clinically, and is able to weight bear with support. Pathological findings from brain biopsy taken at surgery supported a diagnosis of hyperacute ADEM (Fig. 2E–G). The brain tissue was negative in PCR for SARS-CoV-2.

#### Vignette E: sequential para-infectious involvement of central and peripheral nervous systems

A 52-year-old male (Patient 16) presented with a 3-day history of headache, back pain, vomiting and progressive limb weakness. There was bilateral facial and neck weakness, symmetrical upper and lower limb flaccid (proximal > distal) weakness, generalized areflexia, extensor plantar responses and preserved sensation. MRI of the neuroaxis was normal except for gadolinium enhancement of the cervical and lumbar roots (Fig. 2H and L). CSF was acellular, with a raised protein. Nerve conduction studies supported a diagnosis of GBS and he was treated with IVIG. On Day 3 of admission, he deteriorated with increasing weakness, dysphagia, ophthalmoplegia, and lymphopenia. Due to type-2 respiratory failure, he required ventilation. The patient became febrile (38.9°C), with increasing oxygen requirements, and antibiotics were commenced. Chest CT showed bilateral pulmonary infiltrates. SARS-CoV-2 RNA PCR was positive on throat swab, but negative in CSF. On Day 5, he became unresponsive and a repeat brain MRI showed a pattern of T_2_ symmetrical widespread white matter hyperintensities, which progressed further on Day 12 (Fig. 2I–K and M–O). Intravenous methylprednisolone (IVMP, 1 g/day) was given for 5 days, with neurological improvement following treatment on Day 3: eyes opened spontaneously, he could obey commands, mouth words, and move both hands. Two weeks after completion of IVMP the patient was alert, breathing without assistance, talking and able to flex both arms.

### Stroke

Eight patients (Patients 23–30; aged 27–64, six male, two female, four White, two Black, two Asian) had ischaemic stroke in the context of hypercoagulability and a significantly raised D-dimer (>7000 mg/l) in each of the six cases measured. Thrombus was observed in large intra- and extra-cranial vessels in four patients. Four patients had pulmonary thromboembolism (e.g. Patient 27, Vignette F) (Table 4).

#### Vignette F: ischaemic stroke with concurrent pulmonary embolism

**Figure 4:**
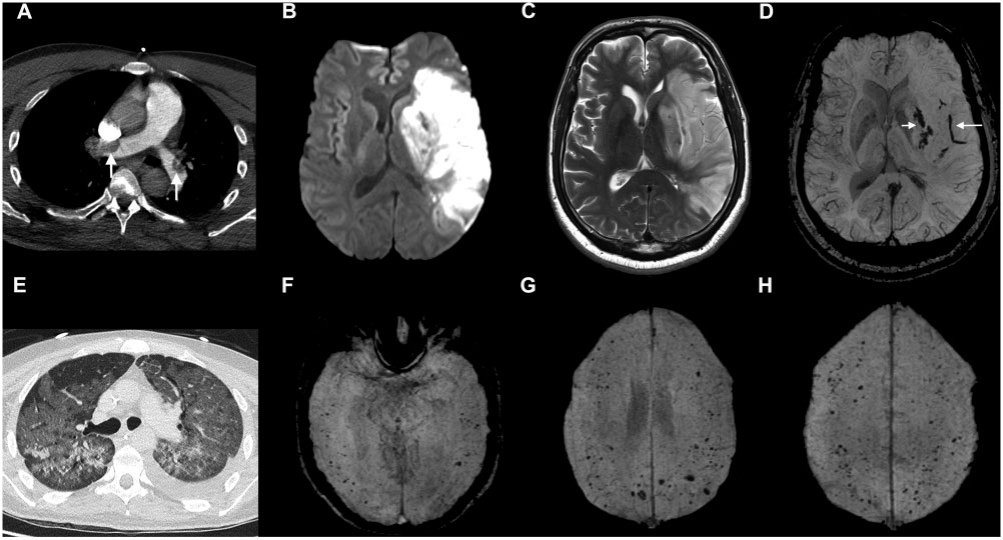
**Imaging from Patient 27, with cerebral infarction and pulmonary thromboembolism (A–D), and Patient 41, with microhaemorrhages (E–H).** (**A**–**D**) Patient 27: CT pulmonary angiogram (**A**) demonstrated large emboli in the right and left pulmonary arteries (arrows). DWI (**B**), T_2_-weighted FSE (**C**) and SWI (**D**) images show restricted diffusion (**B**) and T_2_ hyperintensity (**C**) in the left basal ganglia and cortical territory of left middle cerebral artery. The SWI image (**D**) shows haemorrhagic transformation in the basal ganglia (short arrow) and a long intravascular thrombus in a Sylvain branch of the left middle cerebral artery (long arrow). (**E**–**H**) Patient 41: chest CT (**E**) shows severe COVID-19 pneumonitis. SWI images (**F**–**H**) demonstrate numerous cerebral microbleeds in the temporal, frontal and parietal lobes, predominantly located at the grey/white matter junction.

A 58-year-old male (Patient 27), previously independent in a high-risk occupation for developing COVID-19, presented with acute onset aphasia and dense right-sided weakness. This was preceded by a 2-day history of lethargy and cough. He was found to be drowsy and unresponsive. Brain CT confirmed a proximal middle cerebral artery thrombus and territorial infarct (Fig. 4A–D) with local mass effect. He was transferred to a specialist hospital due to risk of vasogenic oedema leading to malignant middle cerebral artery syndrome. Due to the degree of established infarction neither intravenous thrombolysis nor mechanical thrombectomy were appropriate. CT angiogram also showed a saddle pulmonary embolism, which was managed conservatively with split dose low molecular weight heparin. His condition was critical in the hyperacute period with fluctuating level of consciousness and tachycardia, but stabilized. His D-dimer was in excess of 80 000 µg/l on admission but reduced to 2800 µg/l after 7 days of anticoagulation. Lupus anticoagulant was positive. The patient was discharged to a rehabilitation unit on Day 8.

### Peripheral nervous system

Seven patients (Patients 31–38) with GBS were seen (aged 20–63, all male, five White, one Black, one other), with onset of neurological symptoms from 1 day before to 21 days after typical COVID-19 symptoms. One patient developed a brachial plexopathy onset 2 weeks after COVID-19 symptoms. Of this group, half had definite COVID-19, three requiring intensive care. All GBS patients were treated with IVIG; the patient with brachial plexopathy received corticosteroids. All but two of this group have started to make partial recovery at the time of writing.

### Miscellaneous

The remaining five patients (Patients 39–43) were difficult to categorize. They comprised myelopathy with normal imaging; one patient with bilateral abducens nerve palsy due to intracranial hypertension (pseudo-tumour cerebri) who presented with abdominal pain, diarrhoea and rash and had possible cardiac involvement with COVID-19; a complex paediatric case with a congenital developmental disorder and stable epilepsy who developed non-convulsive status epilepticus; and a patient with a bacterial brain abscess with *Streptococcus intermedius*. One patient, a 27-year-old male with acute myeloid leukaemia, COVID-19 lung disease and seizures with some encephalopathy, demonstrated a significant burden of microhaemorrhages (Patient 41; Fig. 4E–H). He had been treated with gilteritinib as part of his acute myeloid leukaemia therapy.

### Summary of key features

Despite the wide range of initial presentations, with a better appreciation of the five main categories outlined above, the key clinical features and investigations could be proposed as summarized in Table 1. None of the eight patients tested for SARS-CoV-2 PCR in CSF were positive, and none of the autoantibodies seen in autoimmune forms of encephalitis (NMDAR, LGI1) or encephalomyelitis (AQP4, MOG) were detected in serum or CSF samples. Raised D-dimers were, predictably, highly raised in those patients with stroke but were also above normal levels, and occasionally markedly elevated in each of the other groups. Those with encephalopathies improved without specific treatments. The patients with inflammatory CNS diseases were treated with corticosteroids (*n *=* *10) and corticosteroids in combination with IVIG (*n *=* *3) and have had variable outcomes to date, but the follow-up period is still short. One patient with ADEM made some improvement spontaneously without specific treatment. Six of seven patients with GBS had partial response to treatment at the time of writing.

## Discussion

The widespread effects of COVID-19 include neurological disorders but there have been, to date, no detailed clinical reports of their nature (Guan *et al.*, 2020; Helms *et al.*, 2020; Mao *et al.*, 2020; Varatharaj *et al.*, 2020). Our London and regional cohort describes a range of neurological syndromes including encephalopathies, para- and post-infectious CNS syndromes including encephalitis, ADEM with haemorrhage and necrotic change, transverse myelitis, ischaemic stroke and GBS.

The neurological complications of SARS-CoV2 have similarities to those described in the other coronavirus epidemics, specifically severe acute respiratory syndrome (SARS) in 2003, and Middle East acute respiratory syndrome (MERS) in 2012. The cases described in those reports included encephalopathy, encephalitis and both ischaemic and haemorrhagic stroke attributed to hypercoagulability, sepsis and vasculitis, and GBS (Umapathi *et al.*, 2004; Tsai *et al.*, 2005; Kim *et al.*, 2017). However, overall numbers of infected individuals were much smaller, 8000 with SARS and 2500 with MERS, and neurological presentations were therefore few in comparison with those being recognized in the current pandemic.

In a series from Wuhan, 78 of 214 COVID-19 patients, recruited over 4 weeks, developed neurological manifestations. These patients tended to be more severely affected, older and with more comorbidities and, for some, the neurological symptom was the first presentation of COVID-19 (Mao *et al.*, 2020). However, apart from stroke in six patients (2.8%), the neurological features could be due to viral infection (loss of smell and taste) or to the consequences of severe systemic illness in an intensive care setting, such as sepsis and hypoxia. More specific details came from 64 consecutive patients reported by the Strasbourg group (Helms *et al.*, 2020) with agitation in 40/58 (69%), confusion in 26/40 (65%) and corticospinal tract signs in 39/59 (67%). MRI abnormalities were seen in 22 patients with meningeal enhancement, ischaemic stroke and perfusion changes. CSF examination was negative for SARS-CoV-2 in all seven cases tested. There are isolated case reports in the literature of myoclonus (Rábano-Suárez *et al.*, 2020) and demyelination (Varatharaj *et al.*, 2020; Zanin *et al.*, 2020).

Ten of our patients had transient encephalopathies with features of delirium, and psychosis in one. Delirium with agitation is described in case reports and in the larger studies mentioned above, and cognitive dysexecutive syndromes have been reported at discharge (Rogers *et al.*, 2020). While our patients had transient syndromes, detailed neuropsychological testing and follow-up is required to determine the extent of cognitive dysfunction in recovery, and to examine psychiatric and psychological factors (Brown *et al.*, 2020). The underlying mechanisms for the encephalopathy may be multifactorial resulting from the combined or independent effects of sepsis, hypoxia and immune hyperstimultion (‘cytokine storm’) (Mehta *et al.*, 2020).

Two of our cases had a probable autoimmune encephalitis, one with typical clinical features of opsoclonus and myoclonus, and another with typical radiological images as seen in ‘limbic’ encephalitis (Graus *et al.*, 2016). These patients did not have NMDAR, LGI1 or related autoantibodies (Supplementary Table 1). The issue of whether SARS-CoV-2 will trigger a significant number of cases of autoimmune encephalitis, with probable antibody-mediated mechanisms, will become clear in time.

The cluster of cases with an ADEM-like illness warrants close surveillance. ADEM, an immune-mediated demyelinating disorder, is a disease mainly of children (Pohl *et al.*, 2016), with an adult incidence in the UK of 0.23/100 000 (Granerod *et al.*, 2010; Absoud *et al.*, 2015). The nine cases described were accrued over a 5-week period. In Greater London (population 9 million, Office for National Statistics, 2019), we would expect to see this incidence of cases in 5 months, which indicates that COVID-19 is associated with an increased incidence of ADEM. SARS-CoV-2 was not detected in CSF in any of the eight patients tested and the single neuropathological sample obtained did not confirm the presence of SARS-CoV-2 in brain tissue, and was supportive of the diagnosis of ADEM. While we cannot exclude the possibility of direct CNS infection in some cases, without further neuropathological studies or development of accurate CSF viral markers and serological testing, the imaging and clinical features are most supportive of a para- or post-infectious disease mechanism. Long-term follow-up is now required to establish the natural history of the cases that we have identified.

The GBS cases were not unexpected. The temporal relationship between the COVID-19 respiratory illness and the onset of symptoms would be consistent with a post-infectious immune-mediated mechanism. Up to two-thirds of patients with GBS describe an antecedent respiratory or gastroenterological illness. The most common pathogens include *Campylobacter jejuni*, cytomegalovirus, *Mycoplasma pneumonia*, HIV and more recently the Zika virus. The first report of GBS and SARS-CoV-2 from Italy describes five cases of GBS out of a total of 1200 admissions (Toscano *et al.*, 2020). The expected incidence of GBS is 0.6–2.7/100 000/year (Willison *et al.*, 2016) and further epidemiological and mechanistic study is required to determine if there is a true increase in incidence of GBS in COVID-19 patients. Our GBS cases appeared to be similar to conventional GBS patients with respect to clinical presentation, neurophysiology showing demyelinating changes in the majority of patients, CSF parameters and the response to treatment with IVIG.

Stroke associated with a generalized thrombotic predisposition in COVID-19 is of particular interest. Four out of the eight patients had cardiovascular risk factors for stroke including atrial fibrillation. Four also had pulmonary emboli. COVID-19 is associated with a pro-thrombotic state and highly elevated D-dimer levels, and abnormal coagulation parameters have been shown to be associated with poor outcome (Tang *et al.*, 2020). The frequent occurrence of cerebral microbleeds seen in some of the patients, however, was unexpected (Fig. 4). Cerebral microbleeds are usually due to extravasation of red blood cells, and in the context of COVID-19 could be due to endothelial dysfunction related to viral binding to the ACE-2 receptors expressed on endothelial cells. Indeed, a recent report described direct viral infection of the endothelial cell and diffuse endothelial inflammation in multiple organ systems (Varga *et al.*, 2020). The strokes we have encountered with COVID-19 have been severe, and further epidemiological study is required to determine the association between COVID-19 and stroke; randomized trials to determine the optimal use of antiplatelet drugs, low molecular weight heparin and other stroke therapies are required.

Muscle pain and elevated creatinine kinase have been reported as relatively common manifestations of SARS-CoV-2 infection (Chen *et al*., 2020; Guan *et al.*, 2020; Huang *et al.*, 2020) and there are case reports of rhabdomyolysis (Rivas-Garcia *et al.*, 2020). Like other large neurological case series (Varatharaj *et al.*, 2020), we did not observe such cases, but this could reflect referral bias to our MDT, which was set up to discuss the most challenging and severe cases.

Within our cohort of 12 patients with CNS inflammatory syndromes, a range of clinical and radiological presentations were observed, including some suggestive of post-infective ADEM or transverse myelitis and others with more unusual haemorrhagic changes that made classification challenging. A recent MRI study of 37 patients with severe COVID-19 and abnormal brain imaging found three patterns of CNS white matter changes, which could occur in isolation or in combination (Kremer *et al.*, 2020). Pattern 1 featured medial temporal lobe signal abnormalities similar to that seen in viral or autoimmune encephalitis; whereas patterns 2 and 3 featured microhaemorrhages, either in the context of multifocal white matter hyperintense lesions or as separate features, respectively. Whether these patterns represent the same pathology over different timelines, different immunological or other mechanisms, or combinations, is currently unclear, but could have important implications for management decisions, such as the use of steroids, and rehabilitation. In the Kremer *et al.* (2020) study, haemorrhagic lesions correlated with clinical indicators of disease severity. Especially in the intensive care cohort, it can be unclear when brain injury occurs, as imaging is usually only undertaken when a patient is slow to wake after a prolonged period of ventilation.

Histopathological correlates are now emerging for some lesions. Reichard *et al.* (2020) described a case similar to those described in the Kremer *et al.* (2020) study and reported features of both vascular and ADEM-like pathology, with macrophages and axonal injury. Conversely, von Weyhern *et al.* (2020) found lymphocytic panencephalitis and meningitis, and brainstem perivascular and interstitial inflammatory change with neuronal loss as prominent features in six post-mortem patients. In our one case who underwent cranial decompression, brain histology was in keeping with ADEM. Similar to the ADEM-like cases, the GBS cases also largely point to a post-infectious autoimmune mechanism, with most developing the neurological disease within 3 weeks of the documented infection. The risk factors for neurological disease remain unknown, and require further epidemiological study.

The potential mechanisms underpinning the syndromes described include either individually, or in combination, direct viral injury, a secondary hyperinflammation syndrome related to cytokines including IL-6 (Mehta *et al.*, 2020), vasculopathy and/or coagulopathy, post-infectious inflammation including autoantibody production to neuronal antigens, and the effects of a severe systemic disorder with the neurological consequences of sepsis and hypoxia. Evidence of direct viral infection has proved elusive so far with only a few cases with SARS-CoV-2 in CSF reported, and few supportive histopathological features, though clearly further study would be helpful (Reichard *et al.*, 2020, von Weyhern *et al.*, 2020). Elevation of pro-inflammatory cytokines was found to correlate with COVID-19 disease severity (Herold *et al.*, 2020; Huang *et al.*, 2020), and some patients responded to IL-1 or IL-6 blockade (Cavalli *et al.*, 2020; Price *et al.*, 2020); in support of this possible mechanism, transient splenial lesions have been reported in a number of cases, including in children with multisystem inflammatory syndrome (MIS-C), in which elevated cytokines are thought to play a role (Starkey *et al.*, 2017; Abdel-Mannan *et al.*, 2020; Hayashi *et al.*, 2020; Riollano-Cruz *et al*., 2020). Interestingly, some of the clinical features seen in our youngest patient (Patient 39, aged 16 with pseudotumour cerebri with cranial nerve palsies) overlapped with those seen in MIS-C, including gastrointestinal symptoms, rash and cardiac involvement (Supplementary Table 1). Exact mechanisms in each case will be largely speculative until clear clinical, radiological and histological correlates have been drawn; given the breadth of clinical presentations, it is likely that a number or spectrum of these mechanisms are involved.

Collectively, these cases presented a considerable challenge to diagnose with MRI, neurophysiology including EEG, being difficult to obtain in an intensive care setting in addition to the demands of safe nursing and infection control. In many cases, MRI proved essential for making the diagnosis or confident exclusion of abnormalities, especially in patients in the intensive care unit who were ‘slow to wake’. In addition, controversy remains regarding the optimal treatment options including the use of high dose corticosteroids in viraemic, and often lymphopenic patients, and the potential risks of using IVIG for ADEM and GBS, in patients with pro-thrombotic risk factors such as elevated D-dimer levels.

This is a selective and retrospective study, with the limitations associated with this study design, including bias towards severe disease. Nevertheless, the study has allowed a detailed description of the neurological complications seen during and after COVID-19 infection. Further detailed clinical, laboratory, biomarker and neuropathological studies will help elucidate the underlying pathobiological mechanisms of COVID-19 neurological complications. Longitudinal follow-up studies of patients will be necessary to ascertain the long-term neurological consequences of this pandemic.

## Supplementary Material

awaa240_Supplementary_Data

## Abbreviations

ADEM =: acute demyelinating encephalomyelitis
COVID-19 =: coronavirus disease 19
GBS =: Guillain-Barré syndrome
IVIG =: intravenous immunoglobulin
SARS-CoV-2 =: severe acute respiratory syndrome coronavirus 2
