# Supplementary material for: The emerging spectrum of COVID-19 neurology: clinical, radiological and laboratory findings
Source: Brain. 2020 Jul 8;143(10):3104–20. doi: 10.1093/brain/awaa240 (PMC7454352; doi:10.1093/brain/awaa240)
Supplement: awaa240_Supplementary_Data [file awaa240_supplementary_data.pdf]

The emerging spectrum of COVID-19 neurology: clinical, radiological and laboratory findings

Supplementary Table. Demographic, clinical, radiological, laboratory and neuropathological characteristics of forty-three patients with definite, probable and possible COVID-19 infection with central and peripheral neurological syndromes.

Table S1a: Ten patients with encephalopathy (delirium and psychosis)

Table S1b: Twelve patients with inflammatory CNS syndromes (encephalitis, para- and post- infectious) (2 parts)

Table S1c. Demographic, clinical, radiological, laboratory and characteristics of eight individuals with possible and definite COVID-19 infection with ischaemic stroke

Table S1d. Demographic, clinical, radiological, laboratory and characteristics of individuals with possible and definite COVID-19 infection with peripheral neurological syndromes

Table S1e. Five miscellaneous and uncharacterised patients.

Abbreviations used in Tables. CADASIL – Cerebral autosomal dominant arteriopathy with subcortical infarcts and leukoencephalopathy; TIA – transient ischaemic attack; ICU – intensive care unit; N/A – not applicable; Hb – haemoglobin; MCV – mean corpuscular volume; Lymphs – lymphocytes; Neuts – neutrophils; Plt – platelets; ESR – erythrocyte sedimentation rate; CRP – C-reactive protein; NR – no result; TSH – thyroid stimulating hormone; NMDA receptor – N-methyl-D-aspartate receptor; CSF – cerebrospinal fluid; PCR – polymerase chain reaction; CT – computed tomography; MRI – magnetic resonance imaging; CXR – chest X-ray; CTPA – CT pulmonary angiogram; ARDS – acute respiratory distress syndrome; EEG – electroencephalogram; IV – intravenous; IVMP – intravenous methylprednisolone; CPAP – continuous positive airway pressure; ADEM – acute demyelinating encephalomyelitis; MGUS – monoclonal gammopathy of undetermined significance; ANA – anti-nuclear antibodies; ANCA – anti-neutrophil cytoplasmic antibodies; ENA – extractable nuclear antigens; IgG – Immunoglobulin G; GAD – glutamic acid decarboxylase; DPPX - dipeptidyl-peptidase-like protein 6; LGi1 – leucine-rich glioma-inactivated 1; CASPR2 – contactin-associated protein-like 2; MOG – myelin oligodendrocyte glycoprotein; AQP4 – aquaporin-4; FDG PET – fluorodeoxyglucose positron emission tomography; IVIG – intravenous immunoglobulin; JPS – joint position sense; HTLV 1 and 2 – human T-cell leukaemia virus types 1 and 2; EMG – electromyogram; ICP – intracranial pressure; DVT – deep vein thrombosis; PPM – permanent pacemaker; AF – atrial fibrillation, MCA – middle cerebral artery; ACA – anterior cerebral artery; PCA – posterior cerebral artery; mRS – modified Rankin Scale; NHISS – National Institute for Health Stroke Scale; CTA – CT angiogram; PE pulmonary embolism; APTT – activated partial thromboplastin time; IgM – immunoglobulin M; LDL – low density lipoprotein; HDL – high density lipoprotein; GBS – Guillian Barre syndrome; Hep E – hepatitis E; NAD – no abnormality detected; GORD – gastro oesophageal reflux disease; AML – acute myeloid leukaemia; QDS – four times daily; EVD – external ventricular drain

Standard viral PCR – herpes simplex viruses, varicella zoster, enterovirus +/- adenovirus, cytomegalovirus, Epstein-Barr Virus, parechovirus depending upon NHS trust/centre; N-Gene PCR (unvalidated test) for SARS-CoV-2 testing in CSF only where specified.

Supplementary Table. Demographic, clinical, radiological, laboratory and neuropathological characteristics of forty-three patients with definite, probable and possible COVID-19 infection with central and peripheral neurological syndromes.

S1a: Ten patients with encephalopathy (delirium and psychosis)

**Patient**	**1**	**2**	**3**	**4**	**5**	**6**	**7**	**8**	**9**	**10**
**Age (years)**	65	72	59	58	52	39	55	68	50	57
**Gender (M/F)**	F	M	F	M	F	F	F	M	F	M
**Ethnicity**	White	White	Black	Black	White	Asian	White	Black	Black	Black
**Final neurological diagnosis**	Hypoactive delirium	Hypoactive delirium	Delirium	Delirium	Delirium	Delirium	Delirium and psychosis	Hyperactive delirium	Generalised tonic clonic seizures with encephalopathy	Encephalopathy with myelopathy and ataxia
**Past medical history**	CADASIL; previous right occipital stroke; TIA	Bladder cancer; nephrectomy; hypercholesterolaemia	Hypertension; diabetes	Prostate cancer in remission; musculoskeletal back pain	None	Hypertension	None	None	None	Hypertension; diabetes; asthma
**Initial COVID-19 symptoms**	Cough; fever	Confusion; fever	Diarrhoea; vomiting; fever; cough; confusion	Confusion; cough; fever; dysgeusia	Fever; lethargy; confusion	Fever; cough; dyspnoea	Cough; dyspnoea; fever; myalgia; anosmia; hypogeusia	Fever	Cough; household contacts with COVID-19 symptoms	Cough; dyspnoea; fever; myalgia
**Days from onset of COVID-19 symptoms to:**										
1) Hospital admission	15	4	7	4	3	7	1st 14; 2nd 17	1	21	6
2) Onset of neurological symptoms	14	1	3	1	-1	19	17	-4	21	6
3) ICU admission	N/A	6	N/A	N/A	N/A	7	N/A	N/A	N/A	N/A
**Main reason for hospital admis-**	Neurological	Both	Neurological	Neurological	Neurological	Respiratory	1st Respiratory; 2nd Neurological	Neurological	Neurological	Both

**sion (neurological vs respiratory symptoms)**										
**Security of COVID-19 diagnosis (definite, probable, possible)**	Definite	Definite	Definite	Definite	Probable	Definite	Definite	Definite	Probable	Definite
**Severity of COVID-19 infection**	Mild	Critical	Mild	Mild	Mild	Critical	Severe	Mild	Mild	Severe
**Duration of ICU stay (days)**	N/A	13	N/A	N/A	N/A	16	N/A	N/A	N/A	N/A
**Initial neurological symptoms**	Fluctuating confusion; reversal of sleep-wake cycle	Confusion; malaise; loss of appetite	Fluctuating confusion	Confusion; nonsensical speech; repetitive behaviour; disorientation; delusional thoughts; headache	Fluctuating consciousness; delirium	Delirium; hallucinations about experiences in countries not previously visited; reversed sleep/wake cycle	Confusion; agitation; persecutory delusions; visual hallucinations; combative behaviour; headaches	Cognitive impairment; gait disturbance; two falls	Seizures	Double incontinence; progressive lower > upper limb weakness; disorientation; word finding difficulties
**Key neurological signs**	Disorientated to time and place; impaired insight, bradyphrenia; polyminimyoclonus; old left homonymous hemianopia	Cognitive impairment; increased limb tone; brisk reflexes	Fluctuating attention and cognition; bradyphrenia; dyspraxia.	Bilateral intention tremor; heel-shin ataxia	Cognitive impairment; reduced verbal fluency	Cognitive impairment	No focal signs	Disorientation; intermittent agitation; unable to follow commands; speaking a few words only; bilateral extensor plantars	Post-ictal drowsiness and transient disorientation only	Pyramidal tract signs; extensor plantars; mild upper limb dysmetria
**Initial laboratory results at or nearest to time of neurological symptom onset**										
Hb (g/L; 130-170)	130	147	118	162	107	86	113	137	113	172
MCV (fL; 80-99)	84.4	95.4	70.3	79.4	89	89.8	85	73.9	97.2	93.3
Lymphs (x10^9/L; 1.2-3.65)	1.47	0.43	2.31	0.26	0.91	1.16	1.42	1.39	4.48	0.69

Neuts (x10^9/L; 2-7.5)	5.97	4.09	5.09	9.93	5.74	9.52	4.26	3.29	4.65	2.29
Plt (x10^9/L; 150-400)	479	106	276	191	158	599	454	123	379	81
ESR (mm/hr; 1-20)	NR	NR	NR	NR	61	143	NR	NR	NR	NR
CRP (mg/L; 0-5.0)	58.3	64.6	38.1	168.1	56	14.8	37.9	38	8.9	85.5
Fibrinogen (g/L; 1.5-4.0)	NR	NR	NR	NR	4.6	8	7.82	NR	NR	NR
D-Dimer (µg/L; 0-550)	1190	1730	NR	970	NR	2430	1200	NR	NR	1410
Ferritin (µg/L; 30-400)	721	1306	425	3285	393	969	1291	NR	281	1563
Phospholipid antibodies	NR	NR	NR	NR	Negative	NR	NR	NR	NR	NR
Lupus anticoagulant	NR	NR	NR	NR	NR	NR	NR	NR	NR	NR
Other relevant results				Free T4 24.2 (normal range 12-22), TSH 0.07 (0.27-4.20)			Significant negative results: neuronal and NMDA receptor antibodies			
**CSF:**										
White cell count (cells/µL; 0-5)	NR	NR	3	2	NR	NR	<1	3	<1	<1
Protein (g/L; 0.13-0.45)	NR	NR	0.52	Insufficient	NR	NR	0.18	0.4	0.43	0.36

Glucose (mmol/L; 2.2-4.2) (blood glucose) (mmol/L; 3.9-5.8)	NR	NR	NR	3.17 (5.9)	NR	NR	3.5 (5.0)	3.7 (5.3)	NR	3.7 (6.0)
Other			CSF culture and viral PCR negative	CSF culture and viral PCR negative			CSF culture and viral PCR negative	CSF culture and viral PCR negative	Oligoclonal bands negative; CSF culture and viral PCR negative	Matched oligoclonal bands in serum and CSF; CSF culture and viral PCR negative
**Micro/virology**	Blood, urine and sputum cultures negative	Blood and urine cultures negative	Blood cultures negative	Blood and urine cultures negative			Blood and urine cultures negative		Screening for other respiratory viruses negative	Blood, urine, stool cultures all negative
**Imaging**										
Neuroaxis	CT head - no acute changes	CT head - small vessel disease, no acute changes	CT head - small vessel disease	MRI brain normal	CT head - calcification consistent with Fahr's disease; MRI brain – consistent with CT small foci of restricted diffusion in the both centrum semiovale	MRI brain - focal non-specific T2/FLAIR-hyperintensities within globi pallidi bilaterally	MRI brain normal	MRI brain normal	MRI brain normal	MRI brain - mild to moderate small vessel disease, MRI spine - multilevel degenerative disc disease only
Chest	CXR - bilateral infiltrates suggestive of COVID-19	CXR - progressive bilateral, symmetrical nodular infiltrates consistent with COVID-19	CXR – ill-defined bibasal infiltrates and peripheral airspace opacification left lower zone; indeterminate for COVID-19	CXR - normal	CXR – peripheral airspace opacification consistent with COVID-19	CTPA – extensive bilateral ground glass appearance and consolidation consistent with COVID-19 ARDS	CT chest – bilateral subpleural ill-defined ground-glass opacities and interlobular septal thickening consistent with COVID-19	CXR - bibasal infiltrates; CT chest - ground glass changes	CXR - ill-defined peripheral airspace opacification in both lungs, in keeping with probable COVID-19	CXR - Bilateral lower lobe opacities consistent with COVID-19
**Other relevant investigations**										
Neurophysiology							EEG within normal limits	EEG within normal limits		

**Treatment**										
For neurological diagnosis	Supportive	Supportive	Supportive	Supportive	Supportive	Melatonin	Haloperidol; risperidone	1g IVMP 3 days for initial suspicion of autoimmune encephalitis	IV lorazepam acutely for seizures; levetiracetam	Supportive
For COVID-19 infection	None required	CPAP	None required	None required	None required	Intubation and ventilation	Oxygen	None required	None required	Oxygen
**Outcomes**										
Total duration of hospital admission (days)	8	39	14	4	12	20	23	Ongoing	2	12
Outcome at last follow up	Complete recovery; discharged home	Complete recovery of confusion; discharged to rehabilitation centre	Complete recovery; discharged home	Complete recovery; discharged home	Complete recovery; discharged home	Incomplete recovery; ongoing cognitive impairment; discharged home	Significant recovery; improving; discharged home	Ongoing admission; receiving treatment	Complete recovery; discharged home	Complete recovery; discharged home

Table S1b: Twelve patients with inflammatory CNS syndromes (encephalitis, para- and post- infectious) (part 1 of 2)

**Patient**	**11**	**12**	**13**	**14**	**15**	**16**
**Age (years)**	65	66	52	60	59	52
**Gender (M/F)**	F	F	M	M	F	M
**Ethnicity**	Black	White	Asian	Asian	Asian	White
**Final neurological diagnosis**	Possible post-infectious Encephalitis (presumed autoimmune)	Encephalitis	ADEM (with haemorrhage)	ADEM (with haemorrhage)	ADEM (with necrosis and haemorrhage)	ADEM (with haemorrhage) and acute demyelinating poly-radiculoneuropathy
**Past medical history**	Possible Alzheimer's disease; osteoarthritis	Hypertension; hypothyroidism; hysterectomy; osteoarthritis; degenerative spine disease	Asthma	Diabetes; hypertension	Aplastic anaemia; MGUS; treated breast cancer; hypertension; non-alcoholic fatty liver disease; hypercholesterolaemia	None
**Initial COVID-19 symptoms**	Cough; fever; myalgia; diarrhoea	Fever	Cough; myalgia; dyspnoea; hypoxia	Fatigue; myalgia; fever; dyspnoea; hypoxia	Cough; chills; lethargy; myalgia	Fever; hypoxia
**Days from onset of COVID-19 symptoms to:**						
1) Hospital admission	11	1	10	8	10	-4
2) Onset of neurological symptoms	6	1	22	27	10	-6
3) ICU admission	N/A	N/A	10	8	10 (for deterioration in consciousness)	1
**Main reason for hospital admission (neurological vs respiratory symptoms)**	Neurological	Neurological	Respiratory	Respiratory	Neurological	Neurological
**Security of COVID-19 diagnosis**	Definite	Definite	Definite	Definite	Definite	Definite

**Severity of COVID-19 infection**	Severe	Mild	Critical	Critical	Mild	Critical
**Duration of ICU stay (days)**	N/A	N/A	35	35	9	14
**Initial neurological symptoms**	'Shaking'; confusion; hallucinations; reported being 'cross-eyed'; dysarthria	Confusion; single generalised seizure	Slow to wake in ICU	Slow to wake in ICU	Recurrent fleeting episodes of vacant staring and speech arrest; generalised tonic-clonic seizures; headache; low conscious level	Headache; back pain; vomiting; progressive limb weakness
**Key neurological signs**	Perseveration; opsoclonus; convergence spasm; bilateral ocular-facial synkinesis; dysarthria; orofacial apraxia; generalised stimulus-sensitive myoclonus	Low conscious level post-ictally lasting 48 hours; brisk reflexes; extensor plantars	Low conscious level; withdrawal to pain; hyperreflexia and clonus	Low conscious level; opening eyes to voice; withdrawal to pain; right extensor plantar	Left pupil unreactive at nadir; left extensor plantar	Flaccid four limb weakness, proximal > distal; facial and neck weakness; areflexia; extensor plantars, normal sensation; ophthalmoplegia day 3
**Initial laboratory results at or nearest to time of neurological symptom onset**						
Hb (g/L; 130-170)	113	119	86	93	105	155
MCV (fL; 80-99)	95.5	81.5	73.1	96	90	91
Lymphs (x10^9/L; 1.2-3.65)	1.68	0.4	3.07	0.95	0.3	1.07
Neuts (x10^9/L; 2-7.5)	11.02	5.58	13.96	12.79	2.7	5.01
Plt (x10^9/L; 150-400)	759	193	760	370	29 (premorbid baseline 33)	254
ESR (mm/hr; 1-20)	76	NR	NR	NR	NR	22
CRP (mg/L; 0-5.0)	9.1	14	91.5	149	8.1	1
Fibrinogen (g/L; 1.5-4.0)	5.15	3.29	6.5	NR	4.08	NR
D-Dimer (normal range for assay)	1800 µg/L (0-550)	1599 ng/mL (0-230) (day 15, post-IVIG)	80,000 µg/L (0-550)	3330 mg/L (250-750)	2033 mg/L (250-750)	NR
Ferritin (µg/L; 30-400)	261	183	2661	533	98	287

Phospholipid antibodies	NR	NR	NR	NR	NR	NR
Lupus anticoagulant	NR	NR	NR	NR	NR	NR
Other relevant results	ANA positive 1:1280, thyroid peroxidase antibodies positive; paraproteins: 3g IgG lambda, 4g IgG kappa; Significant negatives: neuronal and ganglioside antibodies; antibodies to glycine receptor, GAD, DPPX, NMDA-receptor, LGI1 and CASPR2; ANCA; ENA; complement	Significant negatives: ANA; ANCA; neuronal antibodies; antibodies to LGI1, CASPR2, NMDA receptor GAD, thyroid peroxidase.	ANA positive 1:1280; elevated IgG; Significant negatives: neuronal antibodies, antibodies to NMDA-receptor, LGI1, CASPR2, MOG, AQP4, GAD	ANA and ANCA negative		ANA and ANCA negative
**CSF**						
White cell count (cells/µL; 0-5)	1	3	1	1	4	0
Protein (g/L; 0.13-0.45)	0.22	1.0	0.38	0.22	2.34	1.01
Glucose (mmol/L; 2.2-4.2) (blood glucose) (mmol/L; 3.9-5.8)	3.2 (4.9)	3.5	4.01 (5.37)	5.5	NR	3.6 (6.4)
Other	Matched oligoclonal bands in serum and CSF; neuronal antibodies negative; antibodies to NMDA receptor and GAD negative; CSF culture and viral PCR negative	Oligoclonal bands negative; CSF culture and viral PCR negative, including SARS-CoV-2	Oligoclonal bands negative; Antibodies to NMDA receptor negative; CSF culture and viral PCR negative	CSF culture and viral PCR negative including SARS-CoV-2	Opening pressure 28 cm H20; CSF viral PCR negative including SARS-CoV-2	CSF viral PCR ; negative including SARS-CoV-2
**Micro/virology**	Blood cultures negative	Blood cultures negative		Blood and urine cultures negative; screening for other respiratory viruses negative		
**Imaging**						
Neuroaxis	MRI brain normal	MRI brain - T2 hyperintense signal changes in upper pons, limbic lobes,	MRI brain - Multiple clusters of lesions in the deep cerebral white matter. Cyst-like areas of varied sizes,	MRI brain - Multifocal and confluent areas of signal change in the cerebral hemispheric white matter with	Initial CT head - possible early brainstem swelling, repeat CT day 2 showed hypodensity of brainstem and	MRI brain - Multifocal confluent lesions in internal and external capsules, splenium

		medial thalami and subcortical cerebral white matter. The limbic lobe involvement was symmetrical and included punctate diffusion abnormalities. No micro-haemorrhages or enhancement.	some with haemorrhagic foci and peripheral rims of restricted diffusion were shown within these clusters.	extensive microhaemorrhages in the subcortical regions.	thalami; MRI brain (day 6) Extensive, confluent and largely symmetrical areas throughout brain stem, limbic and insular lobes, superficial subcortical white matter and deep grey matter. Clusters of microhaemorrhages, restricted diffusion and peripheral rim enhancement.	and deep white matter of cerebral hemispheres. Over 5 days, these lesions increased in size and showed multiple microhaemorrhages and extensive prominent medullary veins. Components of brachial and lumbosacral plexus showed increased signal and enhancement without overt swelling.
Chest	CT chest - severe COVID-19 related changes	CT chest suggestive of COVID-19.	CXR - bilateral, lower zone predominant, ill-defined airspace opacification, consistent with COVID-19	CXR - bilateral subpleural airspace infiltrates suggestive of COVID-19	CXR - bibasal atelectasis but no consolidation. Mild pulmonary venous congestion + mild bilateral pleural effusions	CT chest - bilateral infiltrates
Other imaging	FDG PET CT – no malignancy					
**Other investigations**						
Neurophysiology	EEG normal, no correlate for movements		EEG - non-specific cortical dysfunction, mild diffuse encephalopathy			NCS - moderately severe acute demyelinating polyradiculoneuropathy; EEG - encephalopathy, no epileptiform discharges
**Treatment**						
For neurological diagnosis	1g IVMP 3 days, then oral prednisolone taper; levetiracetam; clonazepam	1g IVMP 3 days then oral prednisolone taper, IVIG	Supportive	1g IVMP 3 days	Intubation and ventilation; levetiracetam, aciclovir and ceftriaxone, dexamethasone	Intubation and ventilation; 1g IVMP 5 days; IVIG
For COVID-19 infection	Oxygen	None required	Intubation and ventilation; renal replacement	Intubation and ventilation	Intubation for low conscious level rather than respiratory reasons	Oxygen; intubation and ventilation for type 2 respiratory failure
Other					Platelet infusions for thrombocytopenia	
**Outcomes**						
Total duration of hospital admission (days)	22	28	Ongoing	Ongoing	10	Ongoing
Outcome at last follow up	Incomplete recovery; slow improvement but ongoing myoclonus; discharged home	Incomplete recovery; ongoing cognitive impairment; discharged home	Incomplete recovery; improving	Incomplete recovery; improving	No response to treatment; died	Incomplete recovery; improving; ongoing rehabilitation

Table S1b: Twelve patients with inflammatory CNS syndromes (encephalitis, para- and post- infectious) (part 2 of 2)

**Patient**	**17**	**18**	**19**	**20**	**21**	**22**
**Age (years)**	47	54	60	33	27	48
**Gender (M/F)**	F	F	F	F	F	M
**Ethnicity**	Other	Mixed	Black	White	Asian	White
**Final neurological diagnosis**	ADEM (with haemorrhage)	ADEM	ADEM	ADEM with myelitis	ADEM with myelitis	Post-infectious myelitis
**Past medical history**	asthma	Hypertension; polycystic ovarian syndrome	Hypertension; diabetes	None	None	Hypertension; diabetes
**Initial COVID-19 symptoms**	Cough; fever; dyspnoea	Cough; fever; dysgeusia; truncal rash	Cough; dyspnoea; diarrhoea; fever	Fever	Cough; fever; anosmia; dysgeusia	Cough; dyspnoea; fever
**Days from onset of COVID-19 symptoms to:**						
1) Hospital admission	8	23	4	4	21	1st: day 3; 2nd: day 7; 3rd: day 21
2) Onset of neurological symptoms	8	14	18	2	8	19
3) ICU admission	11 (for deterioration in consciousness)	N/A	4	4 (for deterioration in consciousness)	N/A	N/A
**Main reason for hospital admission (neurological vs respiratory symptoms)**	Neurological	Neurological	Respiratory	Neurological	Neurological	1st Respiratory ; 2nd Respiratory; 3rd Neurological
**Security of COVID-19 diagnosis**	Probable	Probable	Definite	Possible	Probable	Definite
**Severity of COVID-19 infection**	Severe	Mild	Critical	Mild	Mild	Mild
**Duration of ICU stay (days)**	8	N/A	24	34	N/A	N/A

**Initial neurological symptoms**	Subacute left sided numbness and weakness; headache; vomiting; reduced conscious level	Unsteadiness; left sided limb weakness; slurred speech; fatigue; falls	Slow to wake in ICU	Headache; confusion; reduced conscious level	Sensory symptoms in feet and right hand; difficulty with balance and walking	Numbness of hands and feet; band of itching sensation at level of the umbilicus; unsteady gait
**Key neurological signs**	Dense left hemiparesis; reduced sensation on left	Drowsy; slow to respond; dysarthric; trunk and limb ataxia; broad base standing; unable to walk; left-sided pyramidal weakness; bilateral extensor plantars	Extensor posturing of limbs	Low conscious level; brainstem breathing pattern; at worst locked-in; areflexic	Spastic gait, broad based; increased tone with ankle clonus; pyramidal weakness; loss of pinprick and fine touch in feet; no sensory level	Weakness of hip flexion; brisk reflexes; extensor plantars; vibration impaired to costal margins, JPS to ankles, pinprick to T10; sensory ataxia
**Initial laboratory results at or nearest to time of neurological symptom onset**						
Hb (g/L; 130-170)	130	139	68	112	130	157
MCV (fL; 80-99)	84.2	75.1	93.1	93.7	82.4	89.2
Lymphs (x10^9/L; 1.2-3.65)	0.92	2.3	1.74	1.79	1.81	2
Neuts (x10^9/L; 2-7.5)	7.26	6.2	10.12	8.52	2.35	3.37
Plt (x10^9/L; 150-400)	271	345	336	174	249	278
ESR (mm/hr; 1-20)	58	NR	88	43	12	NR
CRP (mg/L; 0-5.0)	34	18.5	169.8	7.6	1.2	<5
Fibrinogen (g/L; 1.5-4.0)	NR	5.7	NR	6.92	NR	3.25
D-Dimer (normal range for assay)	1160 µg/L (0-550)	NR	>8000 ng/mL (<500)	2210 µg/L (0-550)	NR	NR
Ferritin (µg/L; 30-400)	92	NR	521	998	NR	473
Phospholipid antibodies	NR	Negative	NR	Negative	NR	NR
Lupus anticoagulant	NR	Positive	Negative	Positive	NR	NR

Other relevant results	Significant negatives: neuronal antibodies; antibodies to AQP4 and MOG		ANA and ANCA negative	Significant negatives: ANA, ENA, ANCA; antibodies to AQ4, MOG	ANA and ANCA negative	
**CSF:**						
White cell count (cells/µL; 0-5)	Not done (mass effect on imaging).	19 (10% polymorphs, 90% lymphocytes),	<5	54 (95% polymorphs)	4	10 (lymphocytes),
Protein (g/L; 0.13-0.45)	.	0.33	0.36	0.31	0.52	0.7
Glucose (mmol/L; 2.2-4.2) (blood glucose) (mmol/L; 3.9-5.8)	.	4.1 (5.5)	8.4 (12.5)	7.2 (13.5)	3.1 (4.6)	5.6
Other		Oligoclonal bands negative; CSF culture - scanty growth of Staphylococcus capitis - likely contaminant	Oligoclonal bands negative; CSF viral PCR including SARS-CoV-2 negative	Oligoclonal bands negative; antibodies to MOG, AQP4, NMDA receptor, CASPR2 and LGi1 all negative; CSF culture and viral PCR negative including SARS-CoV-2	CSF culture negative; antibodies to AQP4 and MOG pending at time of publication	Matched oligoclonal bands in serum and CSF; CSF culture and viral PCR negative
**Micro/virology**	Blood and urine cultures negative; screen for other respiratory viruses negative; Brain biopsy - viral PCR negative including SARS-CoV-2; mycoplasma PCR negative.		Blood, urine, sputum cultures negative; HTLV1 negative	Tic-borne encephalitis virus, Lyme, returning traveller screen (Porton Down) all negative	HTLV1 serology negative	Blood and urine cultures negative, HTLV 1/2 negative, syphilis negative
**Imaging**						
MRI neuroaxis	Severe right hemispheric vasogenic oedema with a leading edge on contrast imaging. Smaller areas of T2 hyperintense changes in the left hemisphere. Marked mass-effect with 10mm leftwards midline shift, and mild subfalcine herniation	Multiple large lesions with peripheral rim restriction in periventricular white matter of both cerebral hemispheres	Multifocal lesions with diffusion changes in periventricular white matter and corpus callosum.	Initial MR showed multifocal lesions in lower brainstem, medial temporal lobes and cerebral white matter, some of which showed restricted diffusion. 3 days later, the brainstem lesions coalesced and extensive intramedullary lesions with swelling involving grey and white matter of the spinal cord appeared. At 3 week follow-up, these lesions persisted but without swelling or oedema.	Diffuse ill-defined confluent T2 hyperintensity involving the white matter of the cerebral hemispheres, largely along the corticospinal tracts. Small focal area of diffusion change in the left motor cortex. Ill-defined intramedullary lesion without swelling in the conus medullaris.	MRI brain normal; MRI thoracic spine - a patchy area of intramedullary high signal in the dorsal cord primarily at T5-6 and T10-11 and down to the conus with no enhancement with contrast. Felt to be consistent with post-infectious myelitis.
Chest	CTPA – changes at lung bases that could be compatible with COVID-19 infection	CXR - normal	CT chest - bilateral pulmonary infiltrates	CT chest - not typical for COVID-19	NR	CXR - patchy infiltrates; Further CXR - pneumonia

**Other relevant investigations**						
Neurophysiology			EEG – diffuse encephalopathy	EEG - diffuse encephalopathy		Normal nerve conduction studies and EMG
Histology	Brain biopsy - histology consistent with ADEM					
**Treatment given**						
For neurological diagnosis	Intubation for low conscious level; right hemicraniectomy; 1g IVMP 5 days, then oral prednisolone; IVIG.	1g IVMP 3 days, then oral prednisolone	1g IVMP 3 days, then oral prednisolone taper	Intubation for low conscious level; ICP bolt; lumbar drain; 1g IVMP 3 days then oral prednisolone	None	1g IVMP 3 days
For COVID-19 infection	Oxygen	None required	Intubation and ventilation; renal replacement	None required	None required	None required
Other						Antibiotics for secondary bacterial pneumonia
**Outcomes**						
Total duration of hospital admission (days)	Ongoing	Ongoing	Ongoing	Ongoing	0 (Outpatient management)	1st and 2nd: 1 day each; 3rd: 9 days
Outcome at last follow up	Incomplete recovery; improving	Incomplete recovery; improving	Incomplete recovery; improving	Incomplete recovery; improving	Complete recovery; at home	Incomplete recovery; improving; ongoing rehabilitation

Table S1c. Demographic, clinical, radiological, laboratory and characteristics of eight individuals with possible and definite COVID-19 infection with ischaemic stroke

**Patient**	**23**	**24**	**25**	**26**	**27**	**28**	**29**	**30**
**Age at presentation (years)**	61	64	64	53	58	85	73	27
**Gender (M/F)**	M	M	M	F	M	M	M	F
**Ethnicity**	Black	White	White	Asian	Black	White	Asian	White
**Stroke type, observed/implicated mechanism**	Ischaemic right middle cerebral artery occlusion	Ischaemic, vertebral-basilar artery occlusion	Ischaemic bilateral ACA-MCA and MCA-PCA cortical and deep borderzone infarct	Ischaemic, vertebral-basilar artery occlusion	Ischaemic, proximal left middle cerebral artery occlusion	Ischaemic, Left posterior cerebral artery occlusion	Ischaemic basilar artery occlusion	Ischaemic left internal cerebral artery occlusion
**Medical history and risk factors for stroke**	Hypertension; stroke 8 years ago (left-sided weakness); cellulitis; increased body mass index	Nil	Recurrent DVTs (rivaroxaban), Conn Syndrome	Mitral valve replacement (metal valve), atrial fibrillation (on warfarin), heart failure, PPM in situ, hypertension, type 2 diabetes, Grave's disease	Spondylosis	Hypertension, hypercholesterolaemia, atrial fibrillation (on apixaban), ischaemic heart disease, prostate cancer (Gleason Score 4+5)	Gastric carcinoma (resected), benign essential tremor	Nil
**Days from onset of COVID-19 symptoms to:**								
1) Hospital admission	-2	10	7	21	2	10	8	0
2) Onset of neurological symptoms	-2	15	.	22	2	10	8	0
3) ICU admission; duration (days)	Did not go to ICU	Yes - 5	Yes - 13	Yes -8	Did not go to ICU	Did not go to ICU	Did not go to ICU	Yes - 7
**Main reason for hospital admission**	Neurological	Respiratory	Respiratory	Respiratory	Neurological	Neurological	Neurological	Neurological
**Security of COVID-19 diagnosis**	Definite	Definite	Definite	Definite	Probable	Definite	Definite	Probable
**Chest radiograph changes**	CT chest: Bilateral patchy subpleural airspace opacification in both lungs	CXR: Bilateral pulmonary infiltrates	CXR: Bilateral parenchymal airspace infiltrates, more confluent within the lower zones. Bilateral	CXR: Bilateral ground-glass changes and consolidation	CXR: normal CT chest: Lungs clear	peripheral airspace opacities throughout both lungs, worse on right CXR: Bilateral	CXR: Bilateral predominantly peripheral airspace opacities, most confluent at the mid-zones and lung bases	CXR: CT chest: patchy ground glass infiltrates of the left upper lobe. Other indeterminate infiltrates at

			shallow pleural effusions					the level of both lung bases.
**Severity of COVID-19 infection**	Mild	Critical	Critical	Critical	Mild	Mild	Mild	Mild
**Premorbid mRS**	2	0	0	1	0	0	0	0
**Signs and symptoms of stroke**	Dysarthria, left facial droop and left-sided weakness	1st event – left ataxic monoparesis of upper limb; 2nd event 7 days after (despite anticoagulation) - bilateral incoordination and right homonymous hemianopia	Incidental (ICU)	Acute confusion, incoordination, reduced consciousness (GCS 13/15)	Aphasia and right-sided hemiparesis	Dysarthria, right facial droop	Aphasia, right facial droop and right-sided weakness	Dysphasia and left-sided hemiparesis
**Baseline NIHSS**	5	2	.	.	23	6	15	15
**Imaging used for diagnosis**	CT, CTA, MRI	CT, CTA, MRI	CT, CTA, MRI	CT, CTA	CT, CTA, MRI	CT, CTA	CT, CTA, MRI	CT, CTA
**Risk factors for cardio-embolism ¥**	No	No	Yes - AF	Yes - AF	No	Yes - AF	No	No
**Significant carotid stenosis ***	No	No	No	No	No	No	No	No
**Venous thromboembolism**	Yes - PE	Yes - PE	No	No	Yes - PE	No	No	Yes -PE
**Brain imaging**	MRI - Acute infarct in the right corpus striatum. Multiple supra- and infra-tentorial cortical and subcortical microhemorrhages	MRI (1st event): acute and acute left posterior-inferior cerebellar artery territory infarction with microhaemorrhages. 2nd event - 7 days later: bilateral acute posterior cerebral artery territory infarcts despite therapeutic anticoagulation left vertebral artery thrombus	MRI: subacute infarcts within the deep internal borderzones of the cerebral hemispheres bilaterally, and within the left frontal white matter. Background moderate small vessel disease and established cortical infarcts, in arterial borderzone territories.	Non-contrast CT showed acute right parietal cortical and left cerebellar infarct with mass effect and hydrocephalus, despite therapeutic anticoagulantion.	MRI: Extensive evolving left MCA infarct with evidence of petechial haemorrhage and associated mass-effect as described. Persistent occlusion of the left M2 MCA branches	Non-contrast CT: showed hyperdensity consistent with thrombus in the left posterior cerebral artery and acute infarction in the left temporal stem and cerebral peduncle	MRI: acute infarction in the right thalamus, left pons, right occipital lobe and right cerebellar hemisphere. Thrombotic material in the basilar artery and bilateral mild-to-moderate P2 segment stenosis	CT: Extensive acute right middle cerebral artery and right anterior cerebral artery territory infarction. There is local swelling and sulcal effacement with new leftward midline shift of ~4 mm.
**Laboratory results within 24 hours of neurological symptom onset**								

**Hb (g/L; 130-170)**	126	117	70	106	147	128	159	81
**MCV (fL; 80-99)**	94.2	90	100.4	89.1	93.4	85	88	93.5
**Lymphs (x10^9/L; 1.2-3.65)**	1.31	0.53	1.79	2.07	1.49	1.09	1.65	2.16
**Neuts (x10^9/L; 2-7.5)**	6.39	6.65	23.82	18.2	8.87	5.87	6.68	8.96
**Plt (x10^9/L; 150-400)**	408	303	294	328	128	287	632	403
**CRP (mg/L; 0-5.0)**	12.8	280	326.2	97.4	45	161	179.9	119
**Fibrinogen (g/L; 1.5-4.0)**	4.63	9.5	8.82	2.91	3.15	5.3	NR	NR
**D-Dimer (µg/L; 0-550)**	27190	80000	29000	7750	75320	16100	NR	NR
**Prothrombin time (secs; 10-12)**	10.7	11.6	12.6	34.4	12.2	11.3	14.9	11.5
**APTT (secs; 25-37)**	29	35	34	64.7	23	33	30	26
**Cardiolipin antibody**	Negative	IgM (Medium titre)	IgG (low titre)	Negative	Negative	Negative	Negative	Negative
**Lupus anticoagulant**	Positive	Positive	NR	Positive	Positive	Negative	Negative	Positive
**Beta-2 glycoprotein**	Negative	IgM and IgG (low titre)	NR	Negative	Negative	Negative	IgM (low titre)	Negative
**Ferritin (µg/L; 30-400)**	1167	3563	1044	828	NR	NR	NR	NR
**Total cholesterol (mmol/L; 2.5-5.0)**	4.1	2.6	NR	2.2	5	2.9	4.1	6.2
**LDL (mmol/L; 0-3.5)**	2.4	1.4	NR	0.8	3.4	1.1	2.2	3.4
**HDL (mmol/L; 0.9-1.5)**	1	0.5	NR	0.9	0.9	0.9	0.8	1.1
**Triglyceride (mmol/L; 0.4-2.3)**	1.5	1.5	NR	1	1.6	1.9	2.4	3.8

**Glucose (mmol/L; 3.9-5.8)**	6.2	6.9	NR	6.8	6.5	9.4	NR	NR
**Management:**								
**Tissue Plasminogen activator**	No	No	No	No	No	No	Yes	No
**Mechanical Thrombectomy**	No	No	No	No	No	No	No	No
**Antithrombotic therapy**	LMWH	LMWH	LMWH	LMWH	LMWH	aspirin seven days then switched to apixaban	aspirin five days then switched to LMWH	Aspirin 10 days then LMWH
**Outcome Status**	Rehabilitation unit	Rehabilitation unit	Remains static in ICU (day 31)	Died	Rehabilitation unit	Rehabilitation unit	Stroke Unit	Rehabilitation unit

Footnote: ¥ Atrial fibrillation/flutter, Severe LVEF, reduced myocardial wall motion activity, left ventricular mural thrombus - sourced from electrocardiogram, cardiac echocardiogram, and 72 hour holter monitor *significant defined by >50% narrowing. LMWH: low molecular weight heparin; mRS: modified Rankin Scale; NHISS: National Institute for Health Stroke Scale; DVT: Deep Vein Thrombosis; PPM: Permanent Pacemaker; AF: Atrial Fibrillation, MCA; Middle Cerebral Artery; ACA: Anterior Cerebral Artery; PCA: Posterior Cerebral Artery.

Table S1d. Demographic, clinical, radiological, laboratory and characteristics of individuals with possible and definite COVID-19 infection with peripheral neurological syndromes

**Patient**	**31**	**32**	**33**	**34**	**35**	**36**	**37**	**38**
**Age at presentation (years)**	61	57	63	42	60	20	38	60
**Gender (M/F)**	M	M	M	M	M	M	M	M
**Ethnicity**	White	White	Other	White	White	Black	White	White
**Final neurological diagnosis**	GBS	GBS	GBS	GBS	GBS	GBS	GBS	Brachial plexopathy
**Past medical history**	Cluster headache, cervical myelopathy, diabetes, arrhythmia	Hypercholesterolaemia	Depression	Nil	Nil	Nil	Nil	Myeloma; cerebellar stroke
**Initial COVID-19 symptoms**	Cough; fever; malaise; headache	Fever; headache; myalgia	Cough; myalgia; dyspnoea; fevers	Cough, fever dyspnoea, diarrhoea, anosmia	Headache; ageusia; anosmia	Cough, fever, ageusia	Cough, diarrhoea	Cough
**Days from onset of COVID-19 symptoms to:**								
1) Hospital admission	15	11	31	14	0	14	23	17
2) Onset of neurological symptoms	14	6	18	13	-1	10	21	14
3) ICU admission	17	N/A	N/A	16	5	N/A	N/A	N/A
**Main reason for admission (neurological vs respiratory symptoms)**	Neurological	Neurological	Neurological	Neurological	Neurological	Neurological	Neurological	Neurological
**Security of COVID-19 diagnosis (definite, probable, possible)**	Possible	Possible	Possible	Definite	Definite	Possible	Definite	Definite
**Severity of COVID-19 infection**	Mild	Mild	Mild	Mild	Critical	Mild	Mild	Mild
**Duration of ICU stay**	11	NA	NA	17 days	41 days (ongoing)	NA	NA	NA

**Initial neurological symptoms**	Distal limb numbness	Distal limb weakness and numbness	Distal paraesthesia; limb weakness; facial weakness	Distal limb numbness and weakness; dysphagia	Distal limb numbness and weakness	Distal limb numbness and weakness	Distal limb numbness, weakness, clumsiness	Unilateral painless arm weakness and numbness
**Key neurological signs**	Quadriparesis; areflexia	Bilateral facial nerve palsy; bulbar weakness; distal limb weakness; areflexia	Right facial nerve palsy; quadriparesis; areflexia; sensory ataxia	Quadriparesis; areflexia; sensory loss	Quadriparesis; areflexia; sensory loss; dysautonomia; facial and bulbar weakness	Mild distal weakness and areflexia	Mild distal weakness; sensory ataxia	Altered sensation L C6 dermatome; weakness below L elbow
**Initial laboratory results at or nearest to time of neurological symptom onset**								
Hb (g/L; 130-170)	125	146	147	135	166	153	152	85
MCV (fL; 80-99)	95.8	82.9	90	87.7	100.1	95.1	92	107.5
Lymphs (x10^9/L; 1.2-3.65)	2.41	2.35	2.2	1.8	1.46	2.72	1.16	3.53
Neuts (x10^9/L; 2-7.5)	5.81	6.31	3.1	10.7	3.48	2.6	3.14	1.32
Plt (x10^9/L; 150-400)	252	456	199	425	256	244	180	142
CRP (mg/L; 0-5.0)	.	9.9	<5	6	71	<5	<5	NR
Fibrinogen (g/L; 1.5-4.0)	NR	NR	4.1	NR	9.31	1.97	2.76	2.86
D-Dimer (µg/L; 0-550)	NR	420	NR	NR	NR	NR	NR	9100
Ferritin (µg/L; 30-400)	NR	410	450	NR	NR	190	NR	NR
Other relevant results	Hep E serology positive	NAD	NAD	NAD	NAD	NAD	NAD	NAD
**CSF**								
White cell count (cells/µL; 0-5)	<1	12	2	3	2	2	<1	NR
Protein (g/L; 0.13-0.45)	0.3	1.24	0.6	0.5	0.6	0.26	0.9	NR

Glucose (mmol/L; 2.2-4.2) (blood glucose) (mmol/L; 3.9-5.8)	NR	NR	4.0 (4.9)	NR	3.4	2.9 (4.4)	3.7 (5.4)	NR
**Imaging**								
Neuroaxis	CT brain normal	MRI brain and spine normal	MRI brain and spine normal	CT brain normal	MRI brain normal	Nerve root enhancement	MRI brain normal	MRI brain and spine normal
Chest	Normal	Abnormal	Normal	Normal	Normal	Normal	Normal	Midzone opacification consistent with COVID-19
**Other relevant investigations**								
Neurophysiology	Demyelinating with severe axonal loss	Demyelinating	Not done	Demyelinating	Demyelinating	Demyelinating with axonal loss	Demyelinating	Not done
**Treatment**								
For neurological diagnosis	IVIG	IVIG	IVIG	IVIG; mechanical ventilation	IVIG	IVIG	IVIG	IV methyl prednisolone
For COVID-19 infection	Nil	Nil	Nil	Nil	Intubation and ventilation	Nil	Nil	Nil
**Outcomes**								
Total duration of hospital admission (days)	30	35	10	17	46 (ongoing)	5	7 (ongoing)	7
GBS disability score (on discharge)	4	2	2	2	5	2	2	NA

Footnote: 'NR' denotes not tested or no result. GBS: Guillian-Barre syndrome; IVIG: intravenous immunoglobulin NAD: nil abnormal detected. GBS disability score: 0 – healthy, 1 – minor symptoms or signs of neuropathy but capable of manual work/capable of running, 2 – able to walk 5m (across an open space) but incapable of manual work/running, 3 – able to walk with a stick, appliance or support (5m across an open space) 4 – bedridden or chairbound, 5 – requiring assisted ventilation (for any part of the day or night), 6 – dead.

Table S1e. Five miscellaneous and uncharacterised patients

**Patient**	**39**	**40**	**41**	**42**	**43**
**Age (years)**	16	17	27	40	20
**Gender (M/F)**	F	F	M	M	F
**Ethnicity**	Asian	Black	White	Asian	White
**Final neurological diagnosis**	Cranial nerve palsies associated with pseudotumour cerebri	Non-convulsive status epilepticus with widespread cortical MRI changes	Seizures; widespread microhaemorrhages in context of critical COVID-19 infection, hypertensive episodes and new AML on Gilteritinib	Pyogenic [*Streptococcus intermedius*] CNS infection post-COVID-19	Myelopathy, cord ischaemia considered
**Past medical history**	Menorrhagia; normal BMI	Cornelia de Lange syndrome; epilepsy; dysmelia; hypertension; visual disturbance; GORD; Nissen fundoplication; gastrostomy.	Acute myeloid leukaemia (new)	Interstitial keratitis; previous wrist and ankle synovitis	None relevant
**Initial COVID-19 symptoms**	Abdominal pain; diarrhoea; maculopapular rash; headache; sore throat; fever.	Fever; cough; dyspnoea; household contacts	Fever	Fever; cough; shortness of breath; household contacts	Cough; coryza; fatigue; myalgia
**Days from onset of COVID-19 symptoms to:**					
1) Hospital admission	6	3	1	14	5
2) Onset of neurological symptoms	10	26	6	14	6
3) ICU admission	N/A	5	23	14	N/A
**Main reason for hospital admission (neurological vs respiratory symptoms)**	COVID-19 symptoms	Respiratory	Other	Neurological; generally unwell	Neurological
**Security of COVID-19 diagnosis**	Probable	Definite	Definite	Possible	Definite
**Severity of COVID-19 infection**	Severe	Critical	Critical	Mild	Mild
**Duration of ICU stay**	N/A	>50 days ongoing	46	27	N/A

**Initial neurological symptoms**	Diplopia; headache with features of raised ICP.	Seizures	Seizures; weakness; fatigue	Return of fever; drenching night sweats; headache; vomiting; confusion; low conscious level	Headache; leg weakness and numbness; constipation
**Key neurological signs**	Evolving cranial nerve signs; progressive bilateral failure of abduction; weakness of left orbicularis oculi; no optic disc swelling	Low conscious level; possible cortical visual impairment; increased tone and clonus	Mild asymmetric weakness;	Low conscious level; left-sided pyramidal weakness; brisk reflexes	Proximal lower limb weakness; absent lower abdominal and lower limb reflexes; flexor plantars; sensory level T10
**Initial laboratory results at or nearest to time of neurological symptom onset**					
Hb (g/L; 130-170)	102	95	94	94	145
MCV (fL; 80-99)	80.1	91	94	60	84.9
Lymphs (x10^9/L; 1.2-3.65)	1.7	1.1	0.58	1	1.6
Neuts (x10^9/L; 2-7.5)	9.3	8.6	0.74	24	2.8
Plt (x10^9/L; 150-400)	81	135	81	521	238
ESR (mm/hr; 1-20)	NR	NR	NR	103	23
CRP (mg/L; 0-5.0)	294	100	434	248	1.3
Fibrinogen (g/L; 1.5-4.0)	NR	NR	8.59	5.01	4.15
D-Dimer (normal range for assay)	626 ng/mL (0-243)	NR	22900 µg/L (0-550)	4970 µg/L (0-550)	NR
Ferritin (µg/L; 30-400)	NR	1367	6467	1592	NR
Phospholipid antibodies	NR	NR	NR	NR	Negative
Lupus anticoagulant	NR	NR	NR	NR	NR
Other relevant results	ANA and ANCA negative; thyroid function normal	Neuronal and NMDA receptor antibodies negative		ANA negative; Ro antibodies equivocal	ANA and ANCA negative; AQP4 and MOG antibodies negative
**CSF**					

White cell count (cells/µL; 0-5)	Two studies performed (2nd 5 days after 1st) 1st: <1; 2nd: <1	3	Not done	1st: 4500 (95% lymphocytes); 2nd: 13,920 (95% polymorphs); 3rd: 405 (27% mononuclear cells, 73% polymorphs); 4th: 40 (18% mononuclear cells, 82% polymorphs); 5th: 121 (predominantly polymorphs); 6th 20 (75% mononuclear cells, 25% polymorphs)	<1
Protein (g/L; 0.13-0.45)	1st: 0.3; 2nd 0.3	0.91		1st: 2.18; 3rd: 1.03; 4th: 1.42; 5th: 0.62; 6th: 0.54;	0.24
Glucose (mmol/L; 2.2-4.2) (blood glucose) (mmol/L; 3.9-5.8)	1st: 3.1; 2nd: 3.3	4.4		1st: 0.7; 3rd: NR; 4th: 2.65; 5th: 2.97; 6th: 3.81	3.2
Other	Opening pressure: 1st: 39 cm H20; 2nd: 27 cm H20; CSF culture and viral PCR negative	CSF culture and viral PCR negative		Unmatched oligoclonal bands detected in CSF; CSF viral PCR negative, PCR for Streptococcus intermedius detected on CSF from day 5 of admission	Oligoclonal bands negative; CSF culture and viral PCR negative, including SARS-CoV-2
**Micro/virology**	Blood and urine cultures negative; Screening for other respiratory viruses negative				
**Imaging**					
Neuroaxis	Dilated optic nerve sheaths and narrowed but patent transverse sinuses; consistent with raised intra-cranial pressure. No parenchymal changes or thrombosis of the head & neck vessels.	Extensive, symmetrical bilateral signal changes without restricted diffusion involving the cortices of the cerebral and cerebellar hemispheres and the thalami.	MRI brain - Extensive foci of susceptibility artefact predominantly at the grey white junctions of cerebral hemispheres. A few foci are noted in the cerebellum. Minimal associated signal changes but no perilesional oedema or significant mass-effect.	MRI brain - Bilateral ring / laminar enhancing cerebral and cerebellar lesions with diffusion restriction and partial haemorrhagic content, consistent with abscesses and apparent ventriculitis with either intraventricular pus and/or haemorrhage.	MRI brain and spine normal
Chest	CXR - bilateral infiltrates		CT chest - extensive parenchymal changes showing typical appearances of COVID-19 infection	No COVID-19 changes	
**Other relevant investigations**					
Echocardiogram	Mild pericardial effusion, preserved ejection fraction 55%				
**Treatment**					
For neurological diagnosis	Therapeutic lumbar puncture; acetazolamide	Dexamethasone 4g QDS 2 days; methylprednisolone 30mg/kg 3 days, then oral prednisolone taper	Levetiracetam	Intubation and ventilation; various antimicrobials including ceftriaxone, linezolid, meropenem, intrathecal vancomycin; ICP monitoring; right then left	Aspirin 75mg daily

				frontal EVDs; focal excision of brain abscess	
For COVID-19 infection	Colchicine for myocarditis; oxygen	Intubation and ventilation	Intubation and ventilation;	None additional	None required
Other			Chemotherapy for AML - Gilteritinib		
**Outcomes**					
Total duration of hospital admission (days)	18	Ongoing	Ongoing	Ongoing	5
Outcome at last follow up	Incomplete recovery; improv-ing; discharged home	No improvement; static	Incomplete recovery; improv-ing; undergoing rehabilitation	Incomplete recovery; improving	Complete recovery; discharged home

Footnote: CADASIL: Cerebral Autosomal Dominant Arteriopathy with Subcortical Infarcts and Leukoencephalopathy; TIA: transient ischaemic attack; ICU: intensive care unit; N/A: not applicable; Hb: haemoglobin; MCV: mean corpuscular volume; Lymphs: lymphocytes; Neuts: neutrophils; Plt: platelets; ESR: erythrocyte sedimentation rate; CRP: C-reactive protein; NR: no result; TSH: thyroid stimulating hormone; NMDA receptor: N-methyl-D-aspartate receptor; CSF: cerebrospinal fluid; PCR: polymerase chain reaction; CT: computed tomography; MRI: magnetic resonance imaging; CXR: chest X-ray; CTPA: CT pulmonary angiogram; ARDS: acute respiratory distress syndrome; EEG: electroencephalogram; IV: intravenous; IVMP: intravenous methylprednisolone; MGUS: monoclonal gammopathy of undetermined significance; ADEM: acute demyelinating encephalomyelitis; ANA: anti-nuclear antibodies; ANCA: anti-neutrophil cytoplasmic antibodies; ENA: extractable nuclear antigens; IgG: Immunoglobulin G; GAD: glutamic acid decarboxylase; DPPX: dipeptidyl-peptidase-like protein 6; LGI1: leucine-rich glioma-inactivated 1; CASPR2: contactin-associated protein-like 2; MOG: myelin oligodendrocyte glycoprotein; AQP4: aquaporin-4; FDG PET: fluorodeoxyglucose positron emission tomography; IVIG: intravenous immunoglobulin; HTLV 1 and 2: Human T-cell Leukaemia Virus types 1 and 2; EMG: electromyogram; ICP: intracranial pressure; EVDL external ventricular drain; Standard viral PCR – herpes simplex viruses (1 + 2), varicella zoster, enterovirus +/- adenovirus, cytomegalovirus, Epstein-Barr Virus, parechovirus depending upon NHS trust/centre; N-Gene PCR (unvalidated test) for SARS-CoV-2 testing in CSF only where specified.
